# Stable expression of a truncated TLX variant drives differentiation of induced pluripotent stem cells into self-renewing neural stem cells for production of extracellular vesicles

**DOI:** 10.1186/s13287-022-03131-4

**Published:** 2022-09-02

**Authors:** Mingzhi Xu, Gang Chen, Yanan Dong, Shensi Xiang, Miaomiao Xue, Yongxue Liu, Haijing Song, Haifeng Song, Yi Wang

**Affiliations:** 1 State Key Laboratory of Proteomics, National Center for Protein Sciences(Beijing), Beijing Proteome Research Center, Beijing Institute of Lifeomics, Beijing, 102206 China; 2 Anti-Radiation Medical Laboratory, Beijing Institute of Radiation Medicine, Beijing, 100039 China; 3 Emergency Medicine, PLA Strategic Support Force Medical Center, Beijing, 100101 China

**Keywords:** TLX, Induced pluripotent stem cells, Neural stem cells, Long-term culture, Extracellular vesicles

## Abstract

**Background:**

Neural stem cells (NSCs)-derived extracellular vesicles (EVs) possess great potential in treating severe neurological and cerebrovascular diseases, as they carry the modulatory and regenerative ingredients of NSCs. Induced pluripotent stem cells (iPSCs)-derived NSCs culture represents a sustainable source of therapeutic EVs. However, there exist two major challenges in obtaining a scalable culture of NSCs for high-efficiency EVs production: (1) the heterogeneity of iPSC-derived NSCs culture impairs the production of high-quality EVs and (2) the intrinsic propensity of neuronal or astroglial differentiation of NSCs during prolonged culturing reduces the number of NSCs for preparing EVs. A NSCs strain that is amenable to stable self-renewal and proliferation is thus greatly needed for scalable and long-term culture.

**Methods:**

Various constructs of the genes encoding the orphan nuclear receptor NR2E1 (TLX) were stably transfected in iPSCs, which were subsequently cultured in a variety of differentiation media for generation of iNSCs^TLX^. Transcriptomic and biomarker profile of iNSCs^TLX^ were investigated. In particular, the positivity ratios of Sox2/Nestin and Musashi/Vimentin were used to gauge the homogeneity of the iNSCs^TLX^ culture. The iNSCs expressing a truncated version of TLX (TLX-TP) was expanded for up to 45 passages, after which its neuronal differentiation potential and EV activity were evaluated.

**Results:**

Stable expression of TLX-TP could confer the iPSCs with rapid and self-driven differentiation into NSCs through stable passaging up to 225 days. The long-term culture of NSCs maintained the highly homogenous expression of NSC-specific biomarkers and potential of neuronal differentiation. EVs harvested from the TLX-expressing NSCs cultures exhibited anti-inflammatory and neuroprotective activities.

**Conclusions:**

iPSC-derived NSCs stably expressing TLX-TP is a promising cell line for scalable production of EVs, which should be further exploited for therapeutic development in neurological treatment.

**Supplementary Information:**

The online version contains supplementary material available at 10.1186/s13287-022-03131-4.

## Background

Neurodegenerative and cerebrovascular diseases together contribute to the majority of mortality and morbidity worldwide, with a limited number of available therapeutic solutions. A growing body of evidence suggests that neural stem cells (NSCs) possess great potential in addressing the unmet medical needs in treating neurological and neurodegenerative disorders [1–6]. Emerging studies have demonstrated that NSC-derived extracellular vesicles (NSC-EVs) can be a promising therapeutic regimen, especially in regenerative therapies [7–11]. Intravenously or intranasally administered NSC-EVs can efficiently cross the blood–brain barrier (BBB) and primarily target glial cells inhibiting neuroinflammatory responses in the brain. NSC-EVs can also modulate neuronal apoptosis when loaded with therapeutics [12–14]. Hence, NSC-EV-based therapeutic interventions have been proven effective in several preclinical models of neuropathological conditions, including ischemic stroke, spinal cord injury, traumatic brain injury and status epilepticus [15–19].

However, therapeutic applications of NSC-EVs face a significant roadblock in its scalable production. Technically, compared to other types of stem cells (*e.g*., mesenchymal stem cells, MSCs) commonly used for EVs production, NSCs are poorly compatible to industrial-scale productions because of their strong propensity toward differentiation into neuronal or glial cells in less than ten passages [20]. Significant difficulties in maintaining the stemness properties of NSCs in the long-term in vitro culture prevent the practical application of NSCs in the scalable production of EVs. Although fetus-derived primary NSCs can be transfected with the oncogene L/C/V-MYC conferring them with the long-term passaging potential [21–27], the scarcity of donors remains a major limitation. On the other hand, induced pluripotent stem cells (iPSCs)-derived NSCs (iNSCs) are more amenable to the widespread therapeutic application than embryonic stem cell-derived NSCs [28, 29]. The presence of MYC oncogene alone in iNSCs does not suffice to maintain their long-term stemness. Instead, exploiting the intrinsic signaling mechanism of NSCs self-renewal and proliferation could be an efficient alternative to overcome the hurdle [30–34], for example, the orphan nuclear receptor *NR2E1* (TLX), which acts as the master regulator of both self-renewal and proliferation mechanisms in NSCs [35–39]. NSCs expressing the recombinant TLX exhibit increased cellular proliferation and can retain the undifferentiated stage for an indefinite time [38, 40, 41]. Interestingly, whether stable expression of TLX could accelerate the transformation of iPSCs into iNSCs and provide iNSCs with the long-term passaging potential is yet to be revealed. Therefore, we sought to investigate whether stable expression of TLX could potentiate high-efficiency and spontaneous differentiation of iPSCs into iNSCs. Here, we evaluated a series of TLX expression vectors and developed a robust protocol for producing a stable and scalable culture of TLX-expressing iNSCs (iNSCs^TLX^) for efficient production of iNSC-EVs. Our results showed that the stable expression of a truncated version of TLX allowed self-driven differentiation of iPSCs into iNSCs, which can be stably cultured for at least 45 passages. Consistent expansion of iNSCs thus provided a rich source of iNSC-derived EVs with immunomodulatory and neuroprotective activities.

## Materials and methods

### Cells and animals

iPSC line, purchased from Nuwacell Biotechnology Co. Ltd (Anhui, China), was cultured on a Matrigel-coated 6-well plate with ncEpic medium, changing every 24 h. Rat pups were supplied by SPF Biotechnology Co. Ltd. (Beijing, China).

### Generation of TLX overexpressing iPSC lines

Coating of culture plate with Matrigel was carried out according to the manufacturer’s recommendation. Briefly, a 6-well plate was coated with 2 mL Matrigel matrix (Corning Incorporated, New York, USA) diluted 100 times, as previously described [42]. iPSCs were treated with Accutase™ (Thermo Fisher Scientific, Waltham, USA) for 7 min at 37 °C for dissociation. The cell precipitates were suspended in 1 mL ncEpic medium (Nuwacell Biotechnology Co. Ltd., Anhui, China) after centrifuged at 1000 rpm for 5 min. Then cells were added onto a Matrigel-coated 6-well plate at a density of 2 × 10^5^ cells/well and cultured with 2 mL ncEpic medium. On the next day, cells were transduced with lentiviral particles (MOI = 10) for 16 h. After that, cells were selected by selecting with 1 μg/mL of puromycin (Thermo Fisher Scientific, Waltham, USA) for 6 days in a ncEpic medium. EFGP fluorescence was detected by Cytation 5 (BioTek) in selected cells. Lentiviral vector and virus production were performed by OBIO Technology (Shanghai) Corp. Ltd.

### Differentiation and proliferation of TLX overexpressing iPSCs into iNSCs^TLX^

Neural induced differentiation Media (NGD) and neural induction medium (NIM) were prepared according to previous reports [43, 44]. Compound C (Medchem Express LLC, Monmouth, UK, 2.5 μM), FGF2 (Sino Biological Inc., Beijing, China, 20 ng/mL), EGF (Sino Biological Inc., 20 ng/mL), SB431542 (Medchem Express LLC., 10 μM), DMH-1 (Medchem Express LLC., 2 μM), LIF (Sino Biological Inc., 10 ng/mL), LDN193189 (Medchem Express LLC., 0.25 μM) and insulin (Beijing Solarbio Science & Technology Co., Ltd., Beijing, China, 10 ng/mL) were added to medium as described previously [6, 43–48]. iPSCs^TLX^ were seeded onto a matrigel-coated 6-well plate at a density of 2.5 × 10^4^ cells/cm^2^. The medium was changed to a differentiation medium on the next day. On day 6, cells were digested by Accutase™ for 7 min at 37 °C. Then iNSCs^TLX^ were seeded into a vitronectin-coated 6-well plate at a density of 2.5 × 10^4^ cells/cm^2^ and passaged every five days.

### Flow cytometry analysis

Cells were fixed and permeabilized with successive washes in True-Nuclear Transcription Factor buffer (Biolegend Inc., San Diego, USA). Cells were then stained with anti-TLX antibody (Mybiosource, 1:400), Alexa Fluor-421 anti-Vimentin antibody (Biolegend Inc., 1:500), PE anti-Sox2 antibody (Biolegend Inc., 1:100), Brilliant Violet-421 anti-Nestin antibody (Biolegend Inc., 1:100), biotin anti-Mushashi-1 antibody (Biolegend Inc., 1:100) and PE/cy5 anti-streptavidin antibody (Biolegend Inc., 0.3125:100). One million events were collected on CytoFlex (Beckman) and analyzed using FlowJo version 10.5.3.

### Immunofluorescence (IF) staining

Cells were fixed with 4% paraformaldehyde (PFA) for 20 min and permeabilized with 0.1% (v/v) Triton X-100 for 4 min, followed by blocking with 5% of normal goat serum for 1 h and incubated with primary antibodies for 1 h at room temperature (RT). After washing with PBS three times, cells were incubated with respective secondary antibodies for 1 h at RT. Dilutions of primary antibodies used in IF assays were as follows: anti-Nestin antibody (Abcam, San Diego, USA, 1:200), anti-Pax6 antibody (Cell signaling Technology, 1:500), anti-Tuj1 antibody (Abcam, 1:1000), anti-Vimentin antibody (Abcam, 1:1000), anti-Olig2 antibody (Abcam, 1:200), anti-Lmx1a antibody (Abcam, 1:100) and anti-Foxa2 antibody (Abcam, 1:50).

### Western blotting

Cells and EVs were lysed in RIPA lysis buffer (Beyotime, China), and protein content was quantified using the BCA assay (Beyotime, China). Samples were incubated for 10 min at 100 °C in a reducing agent and loading buffer. Concentrations of primary antibodies used in the western blotting were as follows: anti-Flag antibody (Sigma, 1:4000), anti-PTEN antibody (Cell signaling Technology, 1:1000), anti-GFAP antibody (Abcam, 1:1000), anti-CD81 antibody (Abcam, 1:200), anti-CD63 antibody (Abcam, 1:1000) and anti-GAPDH antibody (Abcam, 1:1000).

### Quantitative real-time PCR (qRT-PCR) assay

RNA isolation and cDNA synthesis were performed following the manufacturer’s instructions. cDNA samples were diluted 20-fold with molecular biology grade water. The qRT-PCR assay (Vazyme Biotech Co. Ltd., Nanjing, China) was conducted using ChamQ SYBR qPCR Master Mix (without ROX) (Vazyme Biotech Co. Ltd., Nanjing, China) in CFX96 touch real-time PCR detection system (Biorad). The list of primers used is shown in Additional file 2: Table S10.

### Karyotype identification

Karyotype of cells was identified as described previously [49]. Briefly, cells were incubated with colchicine (Medchem Express LLC.) for 2 h at a confluency of 90%. Accutase™ treated cells were then incubated with KCl containing hypotonic solution for 20 min. Cells were fixed with acetic acid/methanol (3:1) overnight at 4 °C, and subsequently stained and photographed at the department of medical genetics, PKU School of Basic Medical Sciences (Beijing, China).

### RNA sequencing analysis

RNA-seq analysis was performed as described previously [50]. Data analysis was supported by GS Medical Co. Ltd. (Beijing, China). The RNA sequencing data were subjected to Gene Ontology (GO) and “Kyoto Encyclopedia” of Genes and Genomes (KEGG) analyses using DAVID V6.8.

### Production of iNSC-EVs from the iNSCs^TLX−TP^ culture

Cell supernatant was circulated through a hollow fiber TFF column (300 kDa, MWCO) for protein removal and concentration [51]. The iNSC-EVs were collected and stored at -80 °C for further purification by DGUC at 100,000 × *g* for 18 h at 4 °C to obtain a continuous density gradient, as previously described [52]. The final retentate was loaded onto the BIA CIMmultus QA Monolithic Column (BIA Separations) on an ÄKTA Pure 25 chromatography system (Cytiva).

### Nanoflow cytometry

The particle size, concentration and purity of EVs were determined using a NanoFCM cytometer (NanoFCM, China). EVs were diluted with 1 × DPBS to a concentration recommended by the manufacturer for 4000–8000 events/min. Assays for determining the vesicular purity of EV samples were carried out based on the protocol reported previously [53].

### Transmission electron microscopy (TEM)

The TEM was used to examine morphologies of milk EVs. iNSC^TLX−TP^-EV samples (10 μL) were placed on formvar carbon-coated copper grids for 10 min, and excess of suspension liquid was removed by soaking with filter papers. Grids were stained with 10 μL of 3% phosphotungstic acid for 3 min at RT, followed by fixing with 2% glutaraldehyde for 5 min. After drying, samples were observed under a TEM system (JEM-2100; Jeol Ltd., Tokyo, Japan) at 200 kV.

### Quantitative proteomics and miRNA transcriptomics

Three iNSC-EVs protein batches were extracted following the previously reported protocol for proteomics study [54]. Samples were analyzed by LC Tandem MS (LC–MS) method. Only peptides that were exclusively assigned to a protein were used for quantitative analysis. Proteins found in iNSC-EVs were compared against the Vesiclepedia database, and then, the common proteins were subjected to GO analysis using the Functional Enrichment analysis tool [55]. In addition, common proteins were submitted to pathway analysis using Reactome version 78 [56, 57]. RNA-seq of iNSC-EVs miRNA was carried out based on previously reported protocols [58]. The top 40 most abundant miRNAs found in iNSC-EVs (P20-P25) were subjected to KEGG analysis by DIANA-279 miRPath version 3 software [59]. On the other hand, common iNSC-EVs miRNAs also were subject to KEGG analysis by DIANA-279 miRPath after comparing miRNAs among three iNSC-EVs bathes.

### Pharmacological evaluations of iNSC-EVs

Rat primary microglia and astrocytes (RPG) were prepared according to previously reported protocols [60]. Briefly, cerebral hemispheres were removed from 24 h old rat pups. The brain was mashed and filtered through the 70 μm cell strainer. Cells were washed three times with PBS and suspended with fresh media before being plated on a poly-D-lysine-coated 75 cm^2^ flask.

Rat primary neuronal (RPN) cultures were prepared from the whole brain of newborn rats. The brain was cut and placed in trypsin-containing culture media. The mixture was incubated at 37 °C for 20 min before the removal of the enzyme. Cells were then washed three times with PBS and suspended with fresh media before being plated on poly-D-lysine-coated 75 cm^2^ flask. After 4 h, the media was replaced by Neurobasal (Gibco) with 1% B27 (Gibco) and 1% Glutamax-I 100 X (Gibco). Half of the culture medium was changed at 24 h before switching to a 72 h-medium change routine.

On day 9, RPG culture was placed in a shaker incubator at 200 rpm for 2 h to isolate microglia. Astrocytes were obtained after trypsinization of the remaining cells. Glial cells were then remixed at the ratio of 7:1 (astrocytes: microglia). The mixed glial cells were serum-starved for 12 h and then cultured in a medium containing lipopolysaccharide (LPS, sigma, 10 μg/mL) with or without iNSC-EVs (1 × 10^6^ p/mL, 1 × 10^7^ p/mL, 1 × 10^8^ p/mL, 1 × 10^9^ p/mL) for 24 h. Cells were subsequently harvested for the qPCR analysis. And supernatant of RPG was collected and added to RPN for 24 h. RPN cells were digested by 0.25% trypsin for apoptosis detection.

### Statistical analysis

Statistical analysis of the data was conducted using the GraphPad Prism 9 software. Data presented in graphs are mean ± standard deviation (SD). All analyses involved a *t*-test (two-tailed) in identifying significant differences. A *p*-value of 0.05 or less was considered statistically significant (*p < 0.05, **p < 0.01, ***p < 0.001, ****p < 0.0001).

## Results

### Lentiviral transduction of recombinant NR2E1 gene in iPSCs

**Fig. 1:**
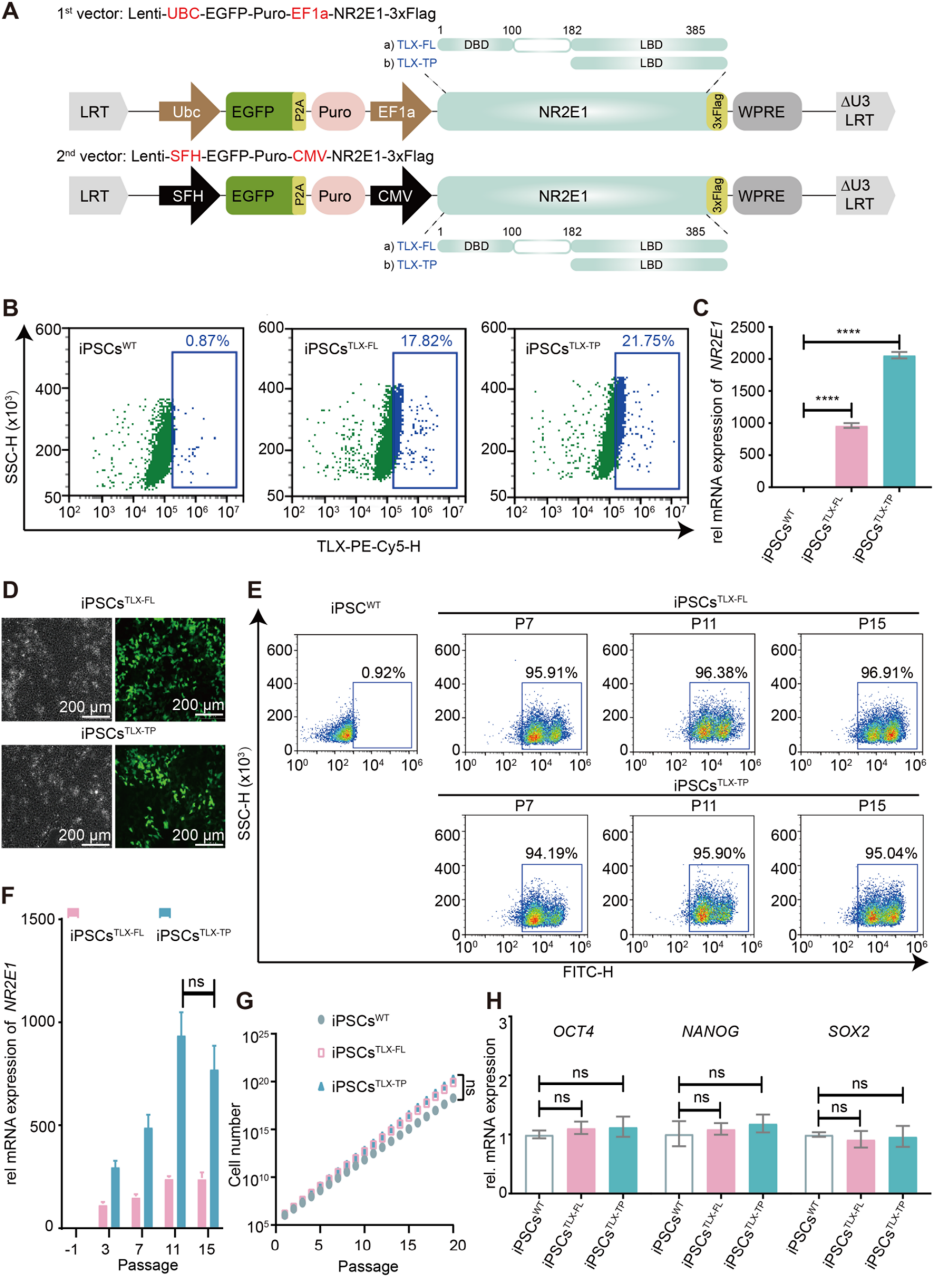
Construction of the iPSC^TLX^ lines. **A** Illustrations of TLX constructs. **B** Flow cytometric analysis of TLX expressions in iPSC^TLX−FL/TP^ lines. **C** Detection of *NR2E1* expression in iPSC^TLX−FL/TP^ lines by qRT-PCR. **D** Morphology of iPSCs^TLX−FL/TP^. **E** Analysis of EGFP expression in different passages of iPSCs^TLX−FL/TP^ by flow cytometry. The expression level and stability of the EGFP gene was not controlled due to the independent expressions of EGFP and the puromycin-resistant genes, hence the two populations of EGFP^+^ cells observed in flow cytometry. **F** Detection of *NR2E1* expression in different passages of iPSCs^TLX−FL/TP^ by qRT-PCR. **G** Proliferation curves of iPSC^TLX−FL/TP^ lines. **H** qRT-PCR analysis of *OCT4*, *SOX2* and *NANOG* expressions in iPSCs^TLX−FL/TP^. The data are representative of three independent experiments and expressed as the means ± SD, ****p < 0.0001, n. s. means no significant difference

TLX consists of two domains (Fig. 1A), namely the DNA-binding domain (DBD, aa1-100) and the ligand-binding domain (LBD, aa182-385). We, therefore, designed gene constructs encoding the full-length protein (TLX-FL) as well as the truncated protein, including only the LBD (TLX-TP) since TLX-TP lacking the DNA-binding ability might lose the activity of regulating critical NSC genes. Each TLX variant was cloned into two expression plasmids carrying either the CMV or the EF1a promoter to evaluate the stability of the transcriptional driving force of these two promoters. For facile distinguishing of the recombinant TLX-FL/TP from intrinsic TLX protein, a 3 × FLAG tag was fused to the C termini of both TLX coding sequences.

Immunoblotting of the iPSCs^TLX−FL^ or iPSCs^TLX−TP^ cells did not detect any expression of the C-terminal FLAG tag or the recombinant TLX protein itself (Additional file 1: Fig. S1), while the flow cytometry showed that about 20% of cells were marginally positive for TLX expressions with fluorescence signal intensities slightly above the baseline (Fig. 1B). EGFP protein expressions driven by the SFH/UBC promoters located upstream of the TLX-3 × FLAG gene were stable and abundant in all transfected cells as expected (Fig. 1D). In stark contrast, transcriptomic and qRT-PCR analyses showed that the transcripts of the recombinant *NR2E1* gene were abundantly present (Fig. 1C, Additional file 2: Table S1), indicating the existence of strong translational and/or post-translational suppression of TLX in iPSCs. Expression of EGFP and the transcription of the recombinant *NR2E1* gene reached a steady state after passage 11 (Fig. 1E and F). Under the iPSCs culture condition, both iPSCs^TLX−FL^ and iPSCs^TLX−TP^ did not exhibit any significant differences in general features, such as clonogenecities, growth rates and cell morphologies (Fig. 1D and G). Expressions of canonical iPSCs biomarkers (*OCT4*, *NANOG* and *SOX2*) were unaffected by the lentiviral transduction of recombinant *NR2E1* gene variants (Fig. 1H), and the rest of the transcriptome profiles of iPSC^TLX−FL^ and iPSC^TLX−TP^ lines were not significantly altered compared to those of iPSC^WT^ lines (Additional file 2: Table S1). Particularly, none of the previously reported target genes of TLX were affected by the transfection.

### Differentiation of iPSCs^TLX−FL/TP^ into iNSCs

**Fig. 2:**
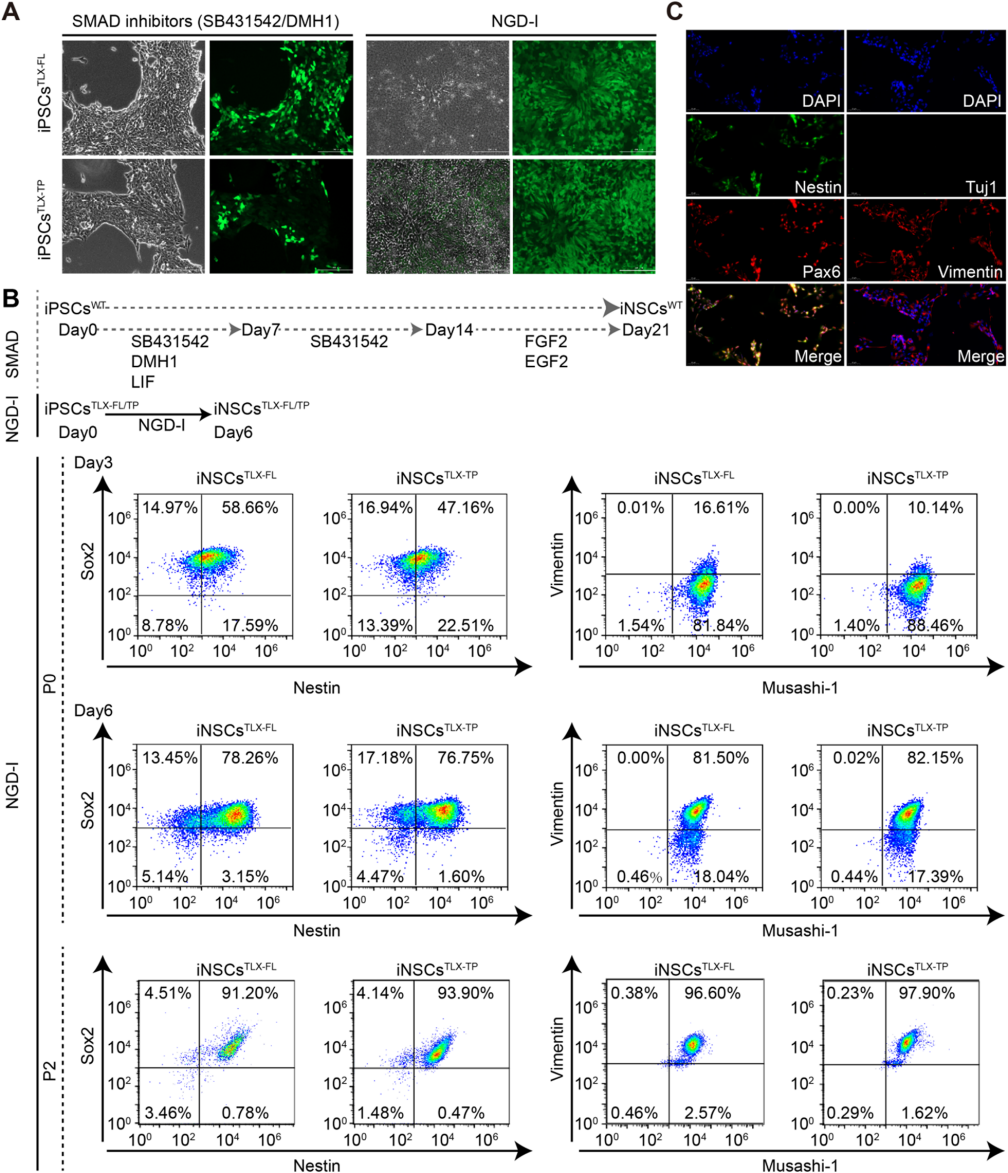
NGD-I medium-induced rapid differentiation of iPSCs^TLX−FL/TP^ into neuroepithelial cells. **A** Morphology of iPSCs^TLX−FL/TP^ cultured in SMAD inhibitor-containing medium or NGD-I medium. Cells cultured in NGD-I showed the emergence of the neural rosette. **B** Schematic representation of differentiation timeline of iPSCs^WT^ or iPSCs^TLX−FL/TP^ lines in SMAD inhibitor-containing medium or NGD-I medium, and the expression of Nestin, Sox2, Vimentin and Musashi-1 in iPSCs^TLX−FL/TP^ cultured in NGD-I on Day 3 and Day 6, and at passage 2. **C** Immunofluorescence (IF) staining of iNSCs^TLX−TP^

**Fig. 3:**
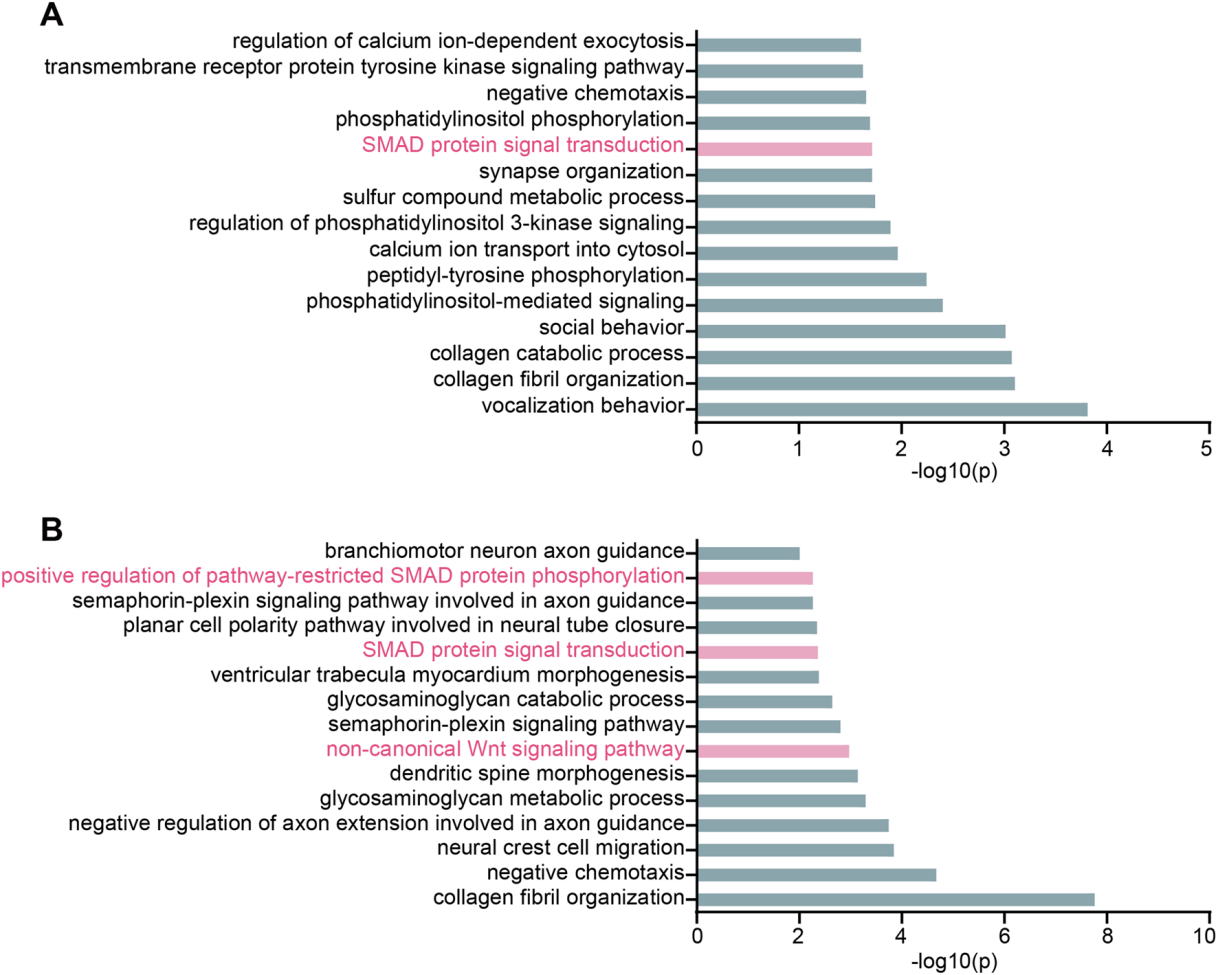
GO analysis of differential gene expressions in (**A)** iNSCs^TLX−FL^ versus iPSCs^TLX−FL^ and (**B)** iNSCs^TLX−TP^ versus iPSCs^TLX−TP^ in terms of biological processes

A variety of previously reported regimens were tested for the efficient differentiation of iPSCs^TLX−FL/TP^ into iNSCs^TLX−FL/TP^, which all led to either rapid cell death or cell cycle arrest (Additional file 2: Table S2). Instead, a basic neural culture medium containing essential supplements such as N2, B27 and insulin could induce rapid differentiation of iPSCs^TLX−FL/TP^ into neuroepithelial cells, with desired morphological changes and the emergence of the neural rosette (Fig. 2A). In contrast to most previously reported NSCs differentiation protocols that normally required 14–21 days to obtain NSC colonies, expressions of NSCs markers like Pax6, Vimentin, Nestin and Musashi-1 emerged as early as Day 3 after the first medium change (Fig. 2B), and 70% of cells were tested positive for NSC biomarkers by Day 6. By the second passage, the Sox2^+^/Nestin^+^ and Vimentin^+^/Musashi-1^+^ cell populations were 91.2% and 96.6%, respectively, for iNSCs^TLX−FL^; and 93.9% and 97.9%, respectively, for iNSCs^TLX−TP^. Interestingly, the addition of any small molecule or protein inducers of NSCs differentiation into this basic medium led to the deterioration of differentiation efficiency or even rapid apoptosis (Additional file 2: Table S2). Overexpression of TLX-FL or TLX-TP might have provided an intrinsic driving force for NSCs differentiation. In the absence of TLX-FL/TP, less than a third of the iPSCs^WT^ cultured in NGD-I medium were positive for NSC biomarkers on Day 6 (Additional file 1: Fig. S2A). Transcriptomic analysis of the iNSCs^TLX−FL/TP^ revealed that the significant regulation of SMAD and Wnt signaling pathways could be involved in the underlying mechanism of this self-driven differentiation (Fig. 3).

### Long-term passaging of iNSCs^TLX−FL/TP^

**Fig. 4:**
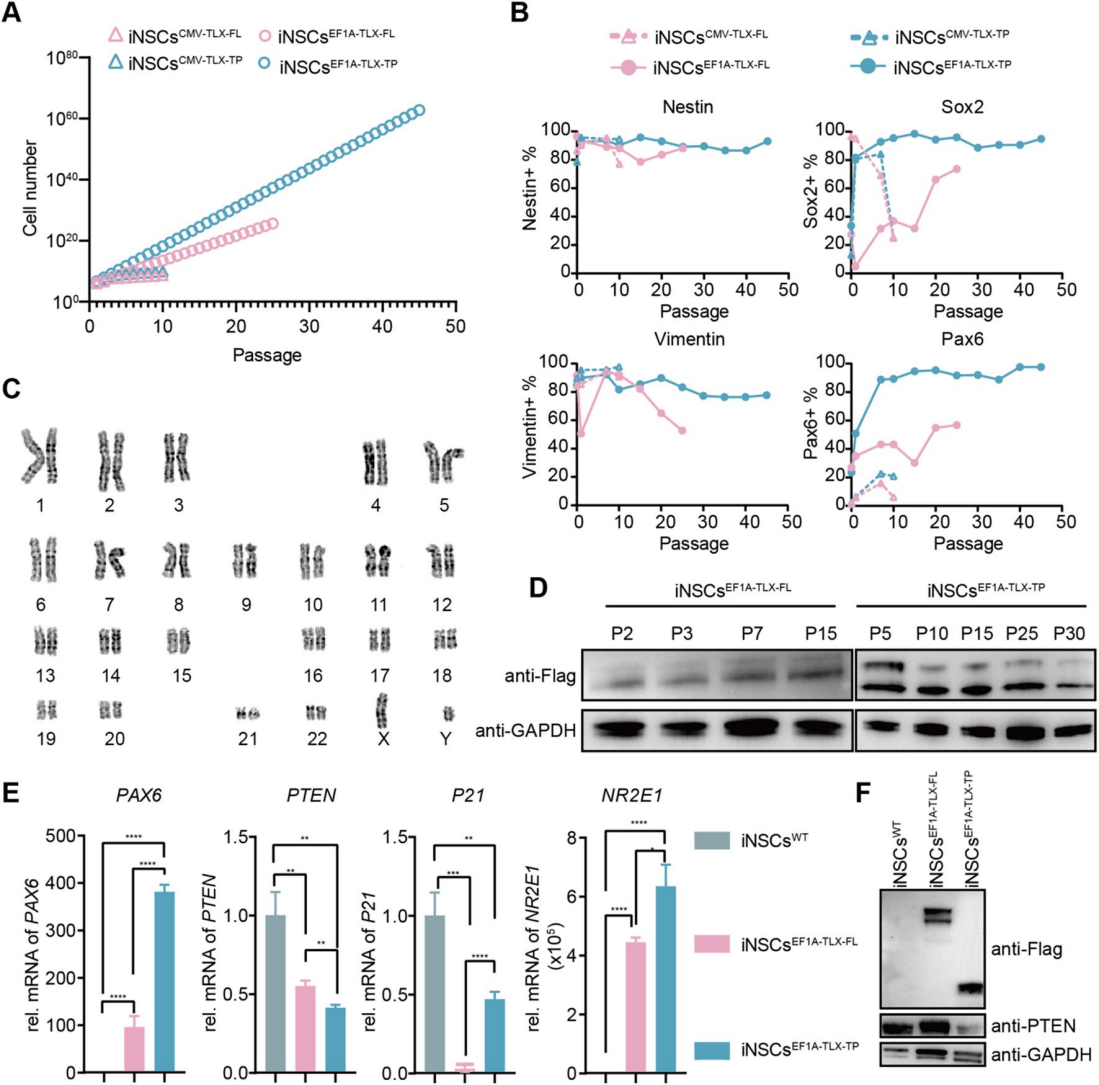
Long-term passaging of iNSCs^TLX−FL/TP^. **A** Proliferations of iNSCs^CMV−TLX−FL/TP^ and iNSCs^EF1a−TLX−FL/TP^. **B** Expressions of Nestin, Sox2, Vimentin and Pax6 in different passages of iNSCs^CMV−TLX−FL/TP^ and iNSCs^EF1a−TLX−FL/TP^ detected by flow cytometry. **C** Karyotype identification showed that the chromosomal integrity of the iNSC^TLX−TP^ lines was unaffected. **D** Expression of Flag in different passages of iNSCs^EF1a−TLX−FL/TP^ detected by immunoblotting. **E** qRT-PCR analysis of expressions of *PAX6*, *PTEN*, *P21* and *NR2E1* genes in iNSCs^EF1a−TLX−FL/TP^. **F** Protein expressions of Flag, PTEN and GAPDH determined by immunoblotting. The data are representative of three independent experiments and expressed as the means ± SD, **p < 0.01, ***p < 0.001

Among different recipes of NSCs expansion media, the commercially available NEM medium supposedly containing TGF-β and GSK3 inhibitors allowed various degrees of expansion of iNSCs^TLX−FL/TP^ (Additional file 2: Table S3). The basic neural culture medium in which iPSCs^TLX−FL/TP^ was differentiated failed to sustain the growth of any iNSC^TLX−FL/TP^ lines, suggesting that the TLX overexpression was sufficient for driving the differentiation but still required TGF-β and GSK3 inhibition for the long-term self-renewal and proliferation. iNSC^TLX−FL/TP^ lines carrying the CMV promoter reached senescence and stopped proliferating by passage 6. The iNSC^TLX−FL^ lines harboring the EF1a promoter proliferated slowly and exhibited halted growth by passage 25, whereas the EF1a-driven iNSCs^TLX−TP^ maintained stable growth that allowed 1:15 passaging (Fig. 4A). The expression levels of NSCs biomarkers remained elevated throughout the 45 passages (225 Days) tested (Fig. 4B), which did not alter the chromosomal integrity of the iNSCs^TLX−TP^ (Fig. 4C). In contrast, iNSCs^WT^ that differentiated from iPSCs^WT^ in the conventional differentiation medium (with SMAD inhibitor) failed to maintain its NSC status and were rapidly adopted the neuronal morphology merely after two passages (Additional file 1: Fig. S3). The expression level of recombinant TLX-TP was decreased in early passages before reaching a stable expression level after passage 10 (Fig. 4D, Additional file 1: Fig. S4), while the level of TLX-FL remained low before reaching the senescence.

In the transcriptome profile of iNSCs^TLX−TP^, a greater number of genes implicated in neural lineage formation were upregulated, compared to that of iNSCs^TLX−FL^, including but not limited to *SOX2*, *WNT7A*, *PAX6*, *ASCL1* and *HES1* (Fig. 4E, Additional file 2: Table S4). BMP4 transcription was markedly downregulated in iNSCs^TLX−TP^ compared to that in iNSCs^TLX−FL^. The CBX family of proteins, which was reported to be the target of the DBD domain of TLX, had differential transcription levels between iNSC^TLX−TP^ and iNSC^TLX−FL^ lines. The overexpression of TLX-TP significantly suppressed the expression of the anti-proliferative protein PTEN, while TLX-FL had a much-diminished effect on the same target (Fig. 4F, Additional file 1: Fig. S5). When the transcriptome profile of iNSCs^TLX−TP^ was compared against that of iNSCs^TLX−FL^, we observed an enrichment of upregulated genes associated with Wnt and Notch signal pathways, which were also vital for the maintenance of the NSC status (Additional file 1: Fig. S6).

### Neuronal and astroglial differentiation of iNSCs^TLX−TP^

**Fig. 5:**
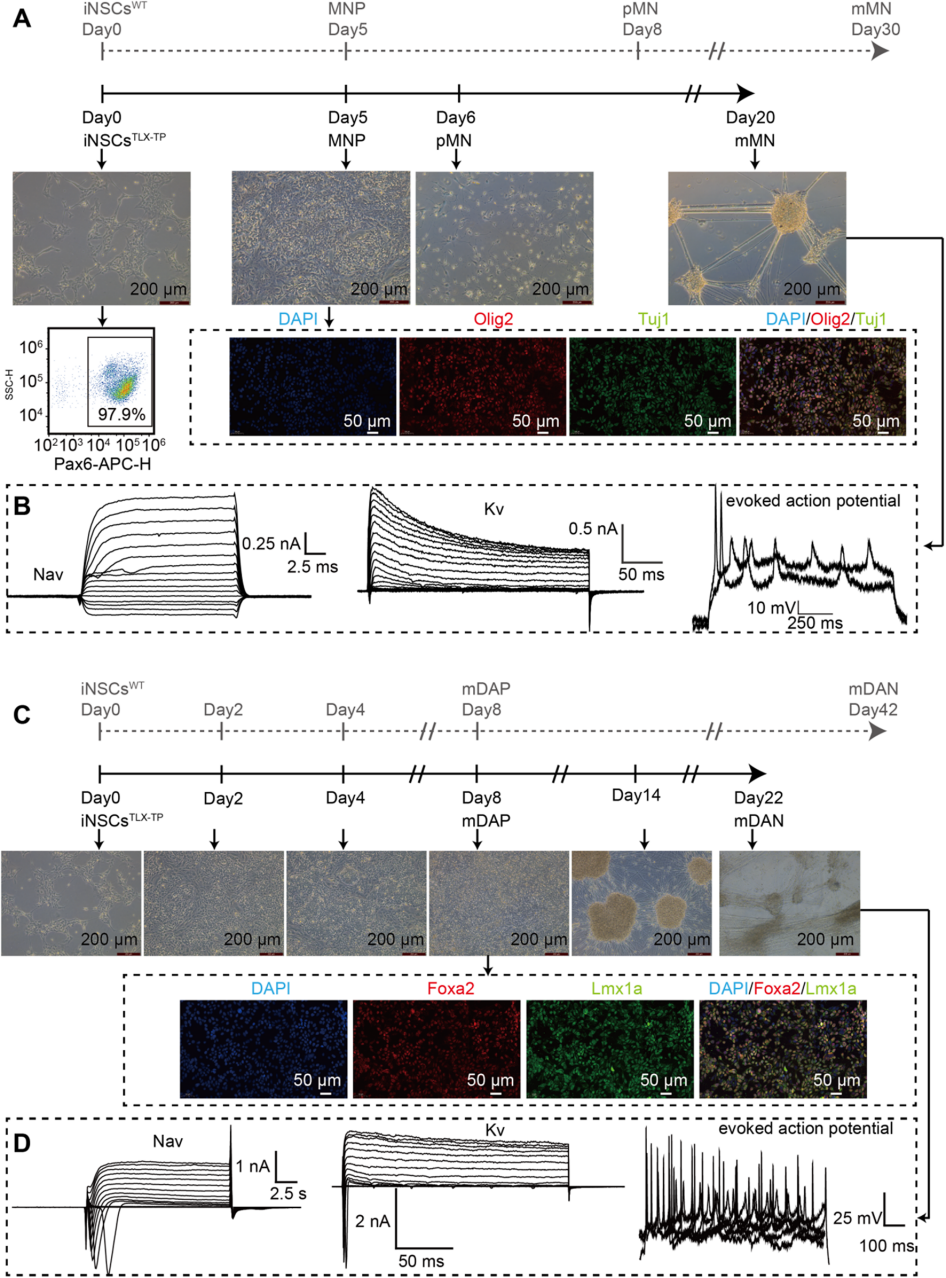
Neuronal differentiation of iNSCs^TLX−TP^. **A** Morphological, flow cytometric and IF staining analyses at different stages of differentiation of iNSCs^TLX−TP^ into motor neurons. **B** Electrophysiological signals, such as Na^+^ and K^+^ channel currents, evoked the action potential of cells on day 20. **C** Morphological and IF staining at different stages of differentiation iNSCs^TLX−TP^ into DAPs. **D** Electrophysiological signals, such as Na^+^ and K^+^ channel currents, evoked the action potential of DAP cells on day 22. MNP: motor neuron progenitor; PMN: postmitotic motor neuron; mMN: mature motor neuron; mDAP: midbrain dopaminergic progenitor; mDAN: midbrain dopaminergic neuron

iNSCs^TLX−TP^ were able to retain their neuronal differentiation potentials even after the long-term passages. In a medium dedicated for motor neuron (MN) or dopaminergic neuron (DA) differentiation, iNSCs^TLX−TP^ exhibited rapid transformation into motor neuron progenitor (MNP) cells or midbrain dopaminergic progenitor (mDAP) cells. Tuj1 and Olig2 expressions were emerged as early as Day 5 after changing the medium from NEM to differentiation medium (Fig. 5A), and electrophysiological signals, such as Na^+^ and K^+^ channel currents and action potential firing, were readily detectable on Day 20 (Fig. 5B). However, differentiation of DAP took only half of the recommended culture period for the differentiated neuronal cells to develop action potential firing capabilities (22 days vs. 42 days, Fig. 5D). DAP markers like LMX1a and Foxa2 were positive on Day 8 (Fig. 5C). In agreement with previous reports that TLX expression favors neuronal differentiation over astroglial differentiation, iNSCs^TLX−TP^ underwent apoptosis 10 days after being cultured in an astrocyte differentiation medium (Additional file 1: Fig. S7).

### Production and characterization of iNSC-EVs from the iNSCs^TLX−TP^ culture

**Fig. 6:**
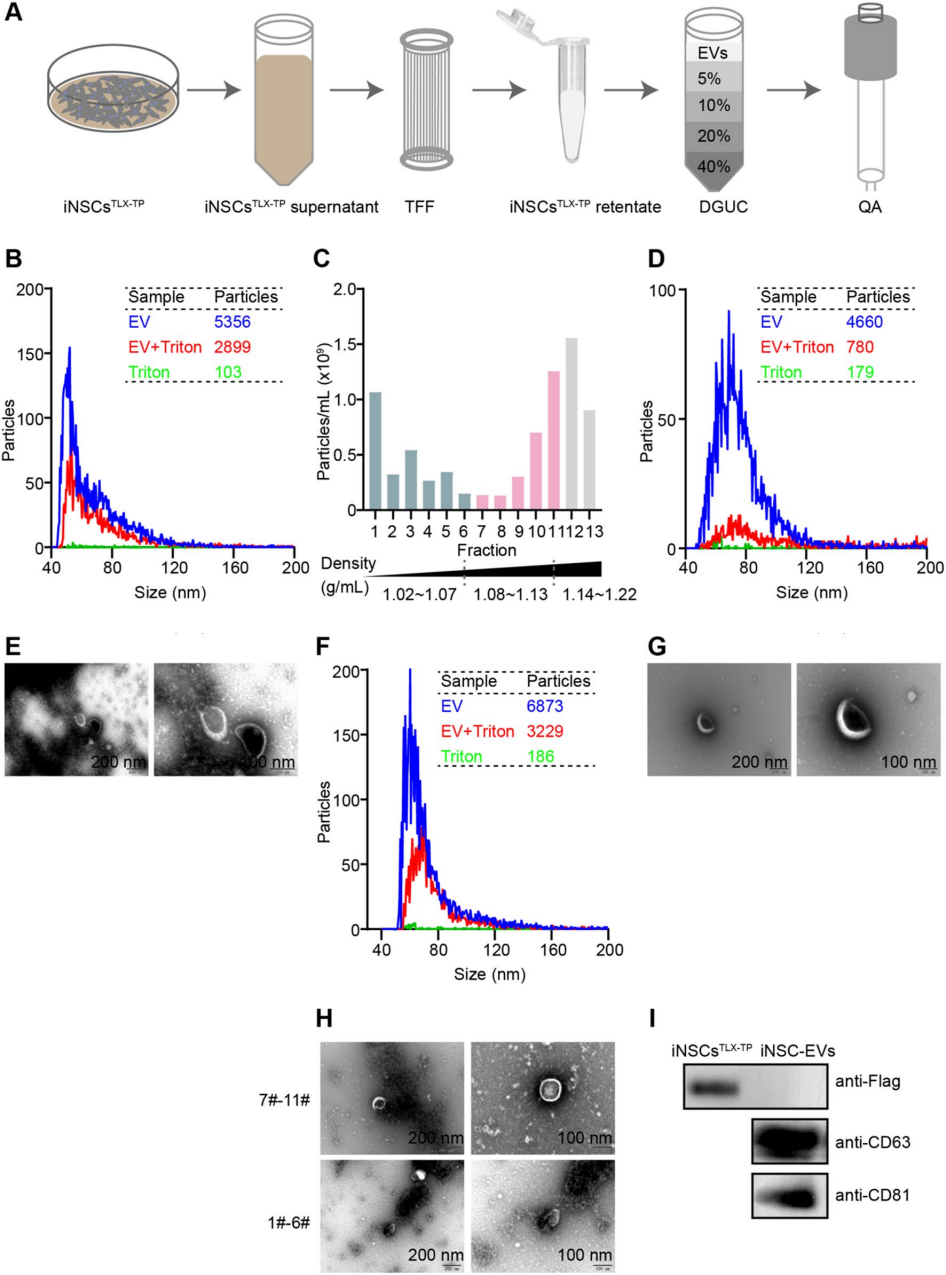
Purification and characterization of iNSC-EVs. **A** Scheme of purification steps of iNSC-EVs. **B** Nanoflow cytometry (nanoFCM) results of iNSC-EVs (blue), iNSC-EVs treated with Triton X-100 (red) and Triton (green) after the TFF step, fractions of DGUC (7–11) and QA (**D**). **C** Concentration of DGUC fraction detected by nanoFCM. TEM results of iNSC-EVs after the TFF step (**E**), QA (**G**) and a fraction (1–6) or fraction (7–11) of DGUC (**H**). **I** Immunoblotting of iNSCs^TLX−TP^ or purified iNSC-EVs for Flag, CD63 and CD81 expressions

The supernatant of the stable subculture of iNSCs^TLX−TP^ was processed through the hollow fiber tangential flow filtration (HF-TFF) column with molecular weight cutoff (MWCO) of 300 kDa for preliminary purification and concentration of EVs. Crude EV suspension was further purified by density gradient ultracentrifugation (DGUC), followed with CIMmultus QA Monolithic column of the semi-purified EV eluate (Fig. 6A). Nanoflow cytometry (NanoFCM) analysis showed that less than half of the particles in the TFF-processed sample were susceptible to Triton X-100-mediated membrane disruption (Fig. 6B), indicative of the significant presence of lipoproteins or protein aggregates (Fig. 6E). NanoFCM and TEM analyses of fractions from DGUC samples showed the presence of large amounts of protein contaminants in fractions 1–6 (Fig. 6C), and vesicles susceptible to Triton X-100 disruption were enriched in fractions 7–11 (Fig. 6F and H). Fractions 7–11 were further purified by CIMmultus QA Monolithic column to 87.1% of purity, and particle sizes were ranged from 40 to 150 nm with a mean value of (64.05 ± 9.24) nm (Fig. 6D and G). The final iNSC-EVs derivative exhibited typical vesicular morphology and contained typical EV surface protein biomarkers, including CD81^+^, CD63^+^, Alix^+^ and TSG101^+^ (Additional file 1: Fig. S8). We also performed western blotting with lysates from iNSC-EVs and iNSCs^TLX−TP^, which confirmed the overexpression of TLX-Flag in iNSCs^TLX−TP^ but not in iNSC-EVs (Fig. 6I, Additional file 1: Fig. S9).

**Fig. 7:**
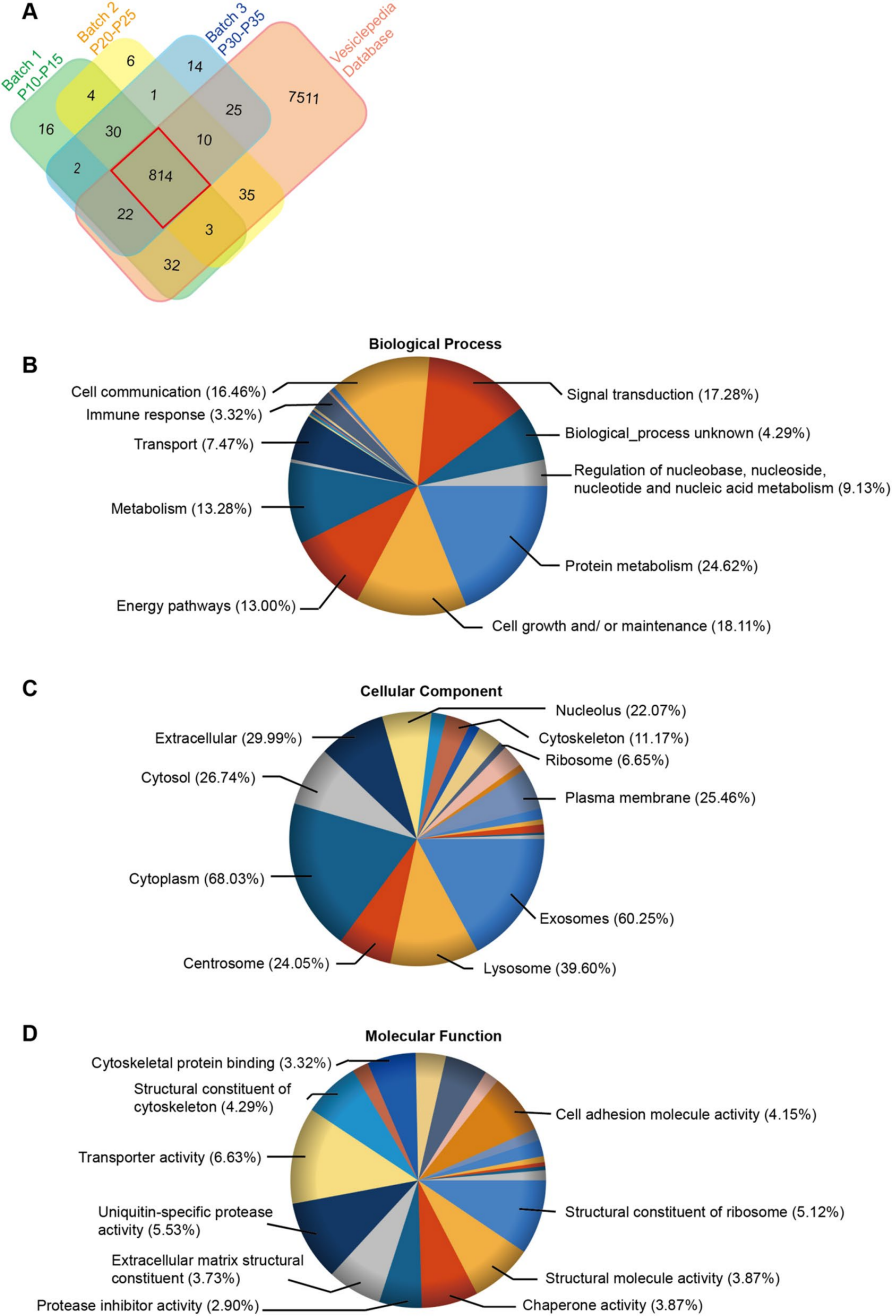
Results of GO analysis of proteins found in iNSC-EVs. **A** A Venn diagram showing that 814 proteins detected in three iNSC-EVs batches were overlapped with the proteins in the Vesiclepedia database. GO analysis of 814 common proteins in biological processes (**B**), cellular component (**C**) and molecular function (**D**)

Over 85% of proteins found in three iNSC-EVs batches were overlapped with proteins in the Vesiclepedia database (Fig. 7A). In total, 814 common proteins in iNSC-EVs were subjected to the Reactome online tool for the related pathway analysis. In total, 748 out of 814 identifiers in the sample were found in Reactome, where 1461pathways were hit at least one of these proteins. The 25 most relevant pathways are presented in Additional file 2: Table S6, where most proteins were associated with the development and regulation of the neural system. GO analysis showed that these proteins could contribute to most biological processes such as protein metabolism, immune response, cell growth and/or maintenance, and cellular communication (Fig. 7B). Many proteins were associated with plasma membrane, exosomes, lysosomes and cytoskeleton (Fig. 7C). The GO analysis based on molecular function revealed that a lot of proteins were involved in cell adhesion molecule activity, cytoskeletal protein binding, transporter activity, structural constituent of ribosome and ubiquitin-specific protease activity (Fig. 7D).

**Fig. 8:**
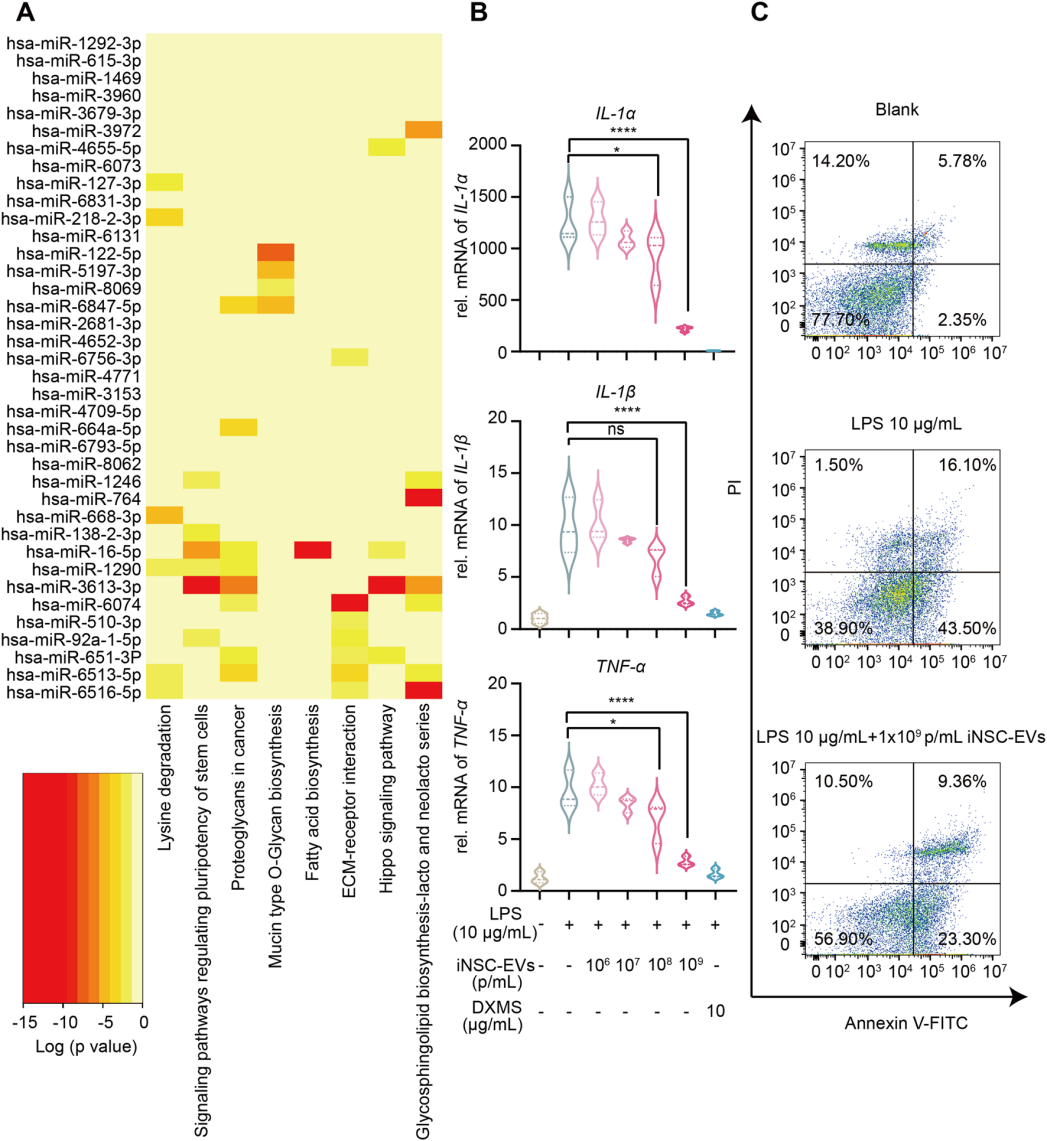
**A** Heatmap of pathway enrichment analysis, including the top 40 most abundant miRNAs found in an iNSC-EVs (P20-P25) batch. **B** Inflammation-related gene (*IL-1α*, *IL-1β* and *TNF-α*) expressions in RPG cells after treating with LPS or LPS plus different concentrations iNSC-EVs for 24 h. **C** Cellular apoptosis analysis of RPN cells after culturing with RPG supernatant for 24 h by flow cytometry. The data are representative of three independent experiments and expressed as the means ± SD, *p < 0.05, ****p < 0.0001, n. s. means no significant difference

The RNA sequencing analysis of iNSC-EVs was performed to determine the composition of miRNAs in EVs. Reads of iNSC-EVs miRNA top 100 are shown in Additional file 2: Table S7. The top 40 miRNAs were analyzed using DIANA-mirPath v.3 and subjected to functional annotation based on the KEGG database. iNSC-EVs were enriched with miRNAs that were mainly implicated in seven signaling pathways related to the central nervous system (CNS), such as signaling pathways regulating pluripotency of stem cells and glycosphingolipid biosynthesis (Fig. 8A, Additional file 2: Table S8). In addition, about 70% of the iNSC-EVs miRNA population were maintained between early and late passages of iNSCs^TLX−TP^ culture, many of which are implicated in various pathways of neural development (Additional file 1: Fig. S10).

We evaluated the biological activity of iNSC-EVs by determining the immunomodulatory effects of iNSC-EVs on rat glial cells. Astrocytes and microglia were mixed in a 7:1 ratio and stimulated with LPS, in the presence or absence of iNSC-EVs. The qRT-PCR analysis showed that iNSC-EVs could suppress the transcription of various cytokine genes in a dose-dependent manner (Fig. 8B). In a neuronal apoptosis model of glial inflammation, rat primary neurons were cultured in the conditioned medium of activated glial cells. The presence of iNSC-EVs significantly reduced the neuronal apoptosis caused by glial cell-secreted cytotoxic cytokines (Fig. 8C).

## Discussion

TLX is a master regulator of NSCs proliferation and self-renewal, while itself is under various types of transcriptional and translational regulations depending on the stage of cell differentiation. Protein levels of the recombinant TLX^FL/TP^ remained low in iPSCs^TLX−FL/TP^, notwithstanding the abundant presence of TLX^FL/TP^ mRNA, indicating the involvement of certain mechanisms related to translational or post-translational suppression of TLX in the iPSC stage. The number of TLX-TP transcripts was greater than those of TLX-FL in both iPSC and iNSC stages (Fig. 1F and 4E). Since the recombinant TLX-FL/TP gene transcripts lacked the intrinsic 3´- and 5´-UTRs of endogenous TLX gene, which was the primary lieu of miRNA-mediated regulation, the gene section encoding the DBD and linker region of TLX might be the target of miRNAs. Indeed, a TLX-targeting miRNA, miR-205-3p, was predicted to bind DBD of TLX and was more abundant in the iPSC stage than in the iNSC stage (Additional file 8: Table S9). Ingredients in the iPSCs culture medium responsible for the maintenance of pluripotency might have contributed to the suppression of TLX mRNA translation, as the switch of medium to basal neuronal culture medium led to increased TLX protein expression and enhanced the driving force for iPSCs to iNSCs differentiation.

Both TLX-TP and TLX-FL exhibited distinct capabilities in stemness maintenance. On the one hand, iNSCs^TLX−FL^ rapidly reached senescence after 25 passages, while iNSCs^TLX−TP^ maintained stable growth up to 45 passages. One possible explanation could be TLX-TP lacked the nuclear export sequence (NES) located in the DBD, leading to the accumulation of TLX-TP in the nucleus, while the overexpression of TLX-FL might have triggered enhanced nuclear export, thus rapidly clearing TLX-FL from the nucleus. Another possible mechanism worth noting could be that LXR and NGFI-B family of nuclear receptors are substrates of ubiquitin E3 ligases, in which the recognition motifs are enriched in the DBD and the linker region [61–63] so that the TLX-TP would be much less susceptible to ubiquitin-mediated degradation compared to TLX-FL, which was supported by immunoblotting results (Fig. 4D). On the other hand, it was rather surprising that TLX-TP lacking the entire DBD, supposedly unable to bring any transcriptional regulators to the vicinity of target genes [37, 39, 64], exhibited superior ability in the NSCs maintenance compared to that of TLX-FL. There could be yet-to-be elucidated mechanisms independent of DBD or TLX-TP that might function as an amplifier of the intrinsic TLX protein. Given that the LBD is responsible for recruiting transcriptional suppressors like Atn1 and HDAC5 [38, 65], TLX-TP could dimerize with the full-length TLX monomer, thus recruiting two copies of transcriptional repressors. In effect, the presence of TLX-TP at least doubled the effective population of intrinsic TLX protein.

Notably, iNSC-EVs are great surrogates for NSC therapy, with the less stringent requirement on storage and transportation. For iPSC-derived cells, in particular, tumorigenicity is always scrutinized by regulatory agencies, as the cells overexpress several oncogenic genes. The iNSCs^TLX−TP^ carrying the recombinant *TLX* gene constituted a considerable risk of oncogenic transformation as well [36], limiting its direct use in cell therapy. Such risk can be effectively mitigated by using iNSC-EVs which were devoid of TLX protein and non-proliferative.

A culture medium with chemically defined ingredients specifically tailored for iNSC-EVs production is still lacking. Our current approach of using the basic neurobasal medium as production medium was suboptimal as cells were starved and underwent apoptosis after 96 h of culture. Vesicle-free culture medium that allows the normal growth of iNSCs^TLX−TP^ could further improve the efficiency and quality of the current iNSC-EVs production system.

## Conclusions

In summary, we developed an iPSC-derived NSC line that allowed the long-term passaging and production of extracellular vesicles with anti-neuroinflammatory activities. Our results not only presented a novel means of generating immortalized NSCs from iPSCs under the intrinsic driving force but also brought to attention the existence of unexplored mechanisms of the nuclear receptor TLX in maintaining NSC self-renewal and proliferation.

## Supplementary Information

**Additional file 1** **Fig. S1**: WB analysis of expressions of Flag and GAPDH in TLX overexpressing HEK293T cells (**A**) and iPSCs (**B**). **Fig. S2**: Induction of differentiation of iPSCs^WT^ into iNSCs^WT^. **A** Detection of expressions of Sox2, Nestin, Vimentin and Musashi-1 in iPSCs^WT^ cultured in NGD-I medium for 6 days by flow cytometry. **B** GO analysis of differential gene expressions between iNSCs^WT^ and iPSCs^WT^ in terms of biological processes. **Fig. S3** Morphologies of iNSCs^WT^ at passages 1 and 2. **Fig. S4**: Expression of Flag and GAPDH in different passages of iNSCs^EF1a-TLX-FL/TP^ detected by WB. **Fig. S5:** Protein expressions of Flag, PTEN and GAPDH determined by WB. **Fig. S6:** **A** GO analysis and **B** KEGG analysis of differential gene expression profiles of iNSCs^TLX-TP^ versus iNSCs^TLX-FL^ in terms of biological processes. **Fig. S7:** Cellular apoptosis during induction of iNSCs^TLX-TP^ differentiation into astrocytes. **Fig. S8:** Immunoblotting of different batches iNSC-EVs for Alix, CD63, CD81, TSG101 and GAPDH expression. **Fig. S9:** Immunoblotting of iNSCs^TLX-TP^ or purified iNSC-EVs for Flag, GAPDH, CD63 and CD81 expressions. **Fig. S10:** The results of transcriptomic analysis of three batches iNSC-EVs samples. **A** A Venn diagram showing that about 70% of the iNSC-EVs miRNA population were shared among the three iNSC-EVs batches. **B** KEGG analysis of 1081 common miRNA.

**Additional file 2** **Table S1**: Comparison of transcriptome profiles of iPSCs^TLX-FL/TP^ and iPSCs^WT^. **Table S2**: List of media tested for iPSCs^TLX-FL/TP^ differentiation. **Table S3:** List of media tested for iNSCs^TLX-FL/TP^ expansion. **Table S4:** Differential gene expressions detected by RNA-sequencing. **Table S5**: NanoFCM analysis results of the media used in iNSCs culture. **Table S6:** The 25 most relevant pathways are overrepresented in the Reactome analysis. **Table S7: **Top 100 abundant miRNAs in iNSC-EVs. Table S8: Enriched KEGG pathways with functional categories related to the CNS. **Table S9:** TLX-targeting miRNAs detected in RNA-sequencing from iPSCs^TLX-FL/TP^ and iNSCs^TLX-FL/TP^. **Table S10**: List of primers.

## Data Availability

The datasets used and/or analyzed during the current study are available from the corresponding author on reasonable request.

## Abbreviations

iPSCs: Induced pluripotent stem cells
NSCs: Neural stem cells
iNSCs: iPSC-derived neural stem cells
SPF: Special pathogen-free
EGFP: Enhanced green fluorescent protein
TEM: Transmission electron microscopy
qPT-PCR: Quantitative real-time PCR
NanoFCM: Nanoflow cytometry
IF: Immunofluorescence
FCM: Flow cytometry
GO: Gene ontology
KEGG: “Kyoto encyclopedia” of genes and genomes
DBD: DNA-binding domain
LPS: Lipopolysaccharide
LBD: Ligand-binding domain
iNSCs^TLX^: TLX-expressing iNSCs
TLX-FL: TLX full-length protein
TLX-TP: TLX truncated protein
NSC-EVs: Neural stem cell-derived extracellular vesicles
BBB: Blood–brain barrier
MSCs: Mesenchymal stem cells
MOI: Multiplicity of infection
NGD: Neural induced differentiation medium
NIM: Neural induction medium
NEM: Neural essential medium
PFA: Paraformaldehyde
RT: Room temperature
RPG: Rat primary glial cells
RPN: Rat primary neuronal cells
CBX: Chromobox
DA: Dopaminergic neuron
mDAP: Midbrain dopaminergic progenitor
MNP: Motor neuron progenitor
MN: Motor neuron
HF-TFF: Hollow fiber tangential flow filtration
MWCO: Molecular weight cutoff
DGUC: Density gradient ultracentrifugation
CNS: Central nervous system
LXR: Liver X receptors
NGFI-B: Nerve growth factor-induced clone B
