# Supplementary material for: Stable expression of a truncated TLX variant drives differentiation of induced pluripotent stem cells into self-renewing neural stem cells for production of extracellular vesicles
Source: Stem Cell Res Ther. 2022 Sep 2;13:436. doi: 10.1186/s13287-022-03131-4 (PMC9438273; doi:10.1186/s13287-022-03131-4)
Supplement: Supplementary file 1 — Additional file 1 Fig. S1: WB analysis of expressions of Flag and GAPDH in TLX overexpressing HEK293T cells (A) and iPSCs (B). Fig. S2: Induction of differentiation of iPSCsWT into iNSCsWT. A Detection of expressions of Sox2, Nestin, Vimentin and Musashi-1 in iPSCsWT cultured in NGD-I medium for 6 days by flow cytometry. B GO analysis of differential gene expressions between iNSCsWT and iPSCsWT in terms of biological processes. Fig. S3 Morphologies of iNSCsWT at passages 1 and 2. Fig. S4: Expression of Flag and GAPDH in different passages of iNSCsEF1a-TLX-FL/TP detected by WB. Fig. S5: Protein expressions of Flag, PTEN and GAPDH determined by WB. Fig. S6: A GO analysis and B KEGG analysis of differential gene expression profiles of iNSCsTLX-TP versus iNSCsTLX-FL in terms of biological processes. Fig. S7: Cellular apoptosis during induction of iNSCsTLX-TP differentiation into astrocytes. Fig. S8: Immunoblotting of different batches iNSC-EVs for Alix, CD63, CD81, TSG101 and GAPDH expression. Fig. S9: Immunoblotting of iNSCsTLX-TP or purified iNSC-EVs for Flag, GAPDH, CD63 and CD81 expressions. Fig. S10: The results of transcriptomic analysis of three batches iNSC-EVs samples. A A Venn diagram showing that about 70% of the iNSC-EVs miRNA population were shared among the three iNSC-EVs batches. B KEGG analysis of 1081 common miRNA. [file 13287_2022_3131_MOESM1_ESM.docx]

Stable expression of a truncated TLX variant drives differentiation of induced pluripotent stem cells into self-renewing neural stem cells for production of extracellular vesicles

Mingzhi Xu^a^ , Gang Chen^a^ , Yanan Dong^a^ , Shensi Xiang^a^, Miaomiao Xue^a^ , Yongxue Liu^b^, Haijing Song^c*^, Haifeng Song^a*^, Yi Wang^a*^

**Additional file 1**


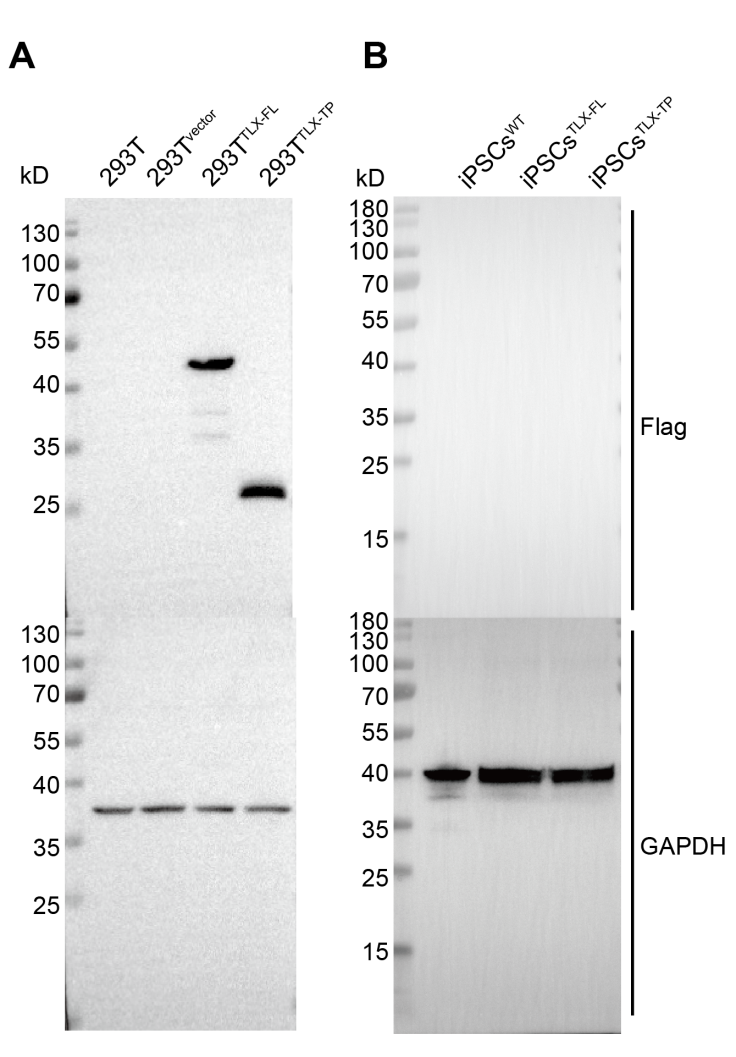


**Fig. S1** WB analysis of expressions of flag and GAPDH in TLX over-expressing HEK293T cells (A) and iPSCs (B).


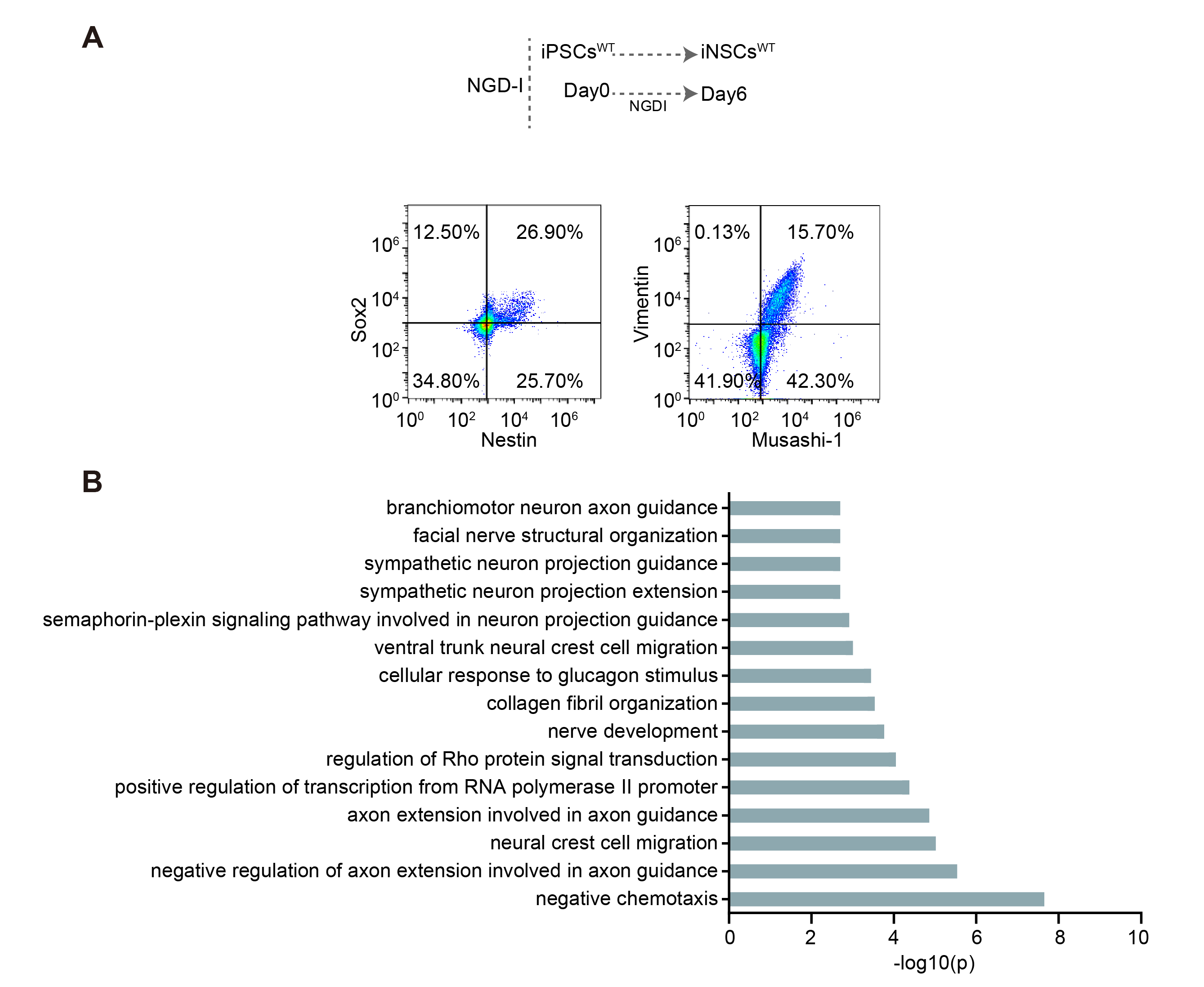


**Fig. S2** Induction of differentiation of iPSCs^WT^ into iNSCs^WT^. A) Detection of expressions of Sox2, Nestin, Vimentin, and Musashi-1 in iPS^WT^ cells cultured in NGD-I medium for 6 days by flow cytometry. B) GO analysis of differential gene expressions between iNSCs^WT^ and iPSCs^WT^ in terms of biological processes.


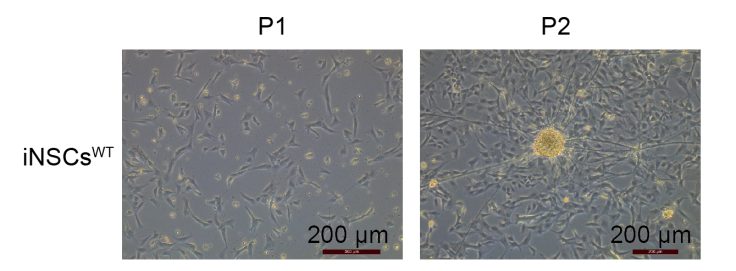


**Fig. S3** Morphologies of iNSCs^WT^ at passages 1 and 2.


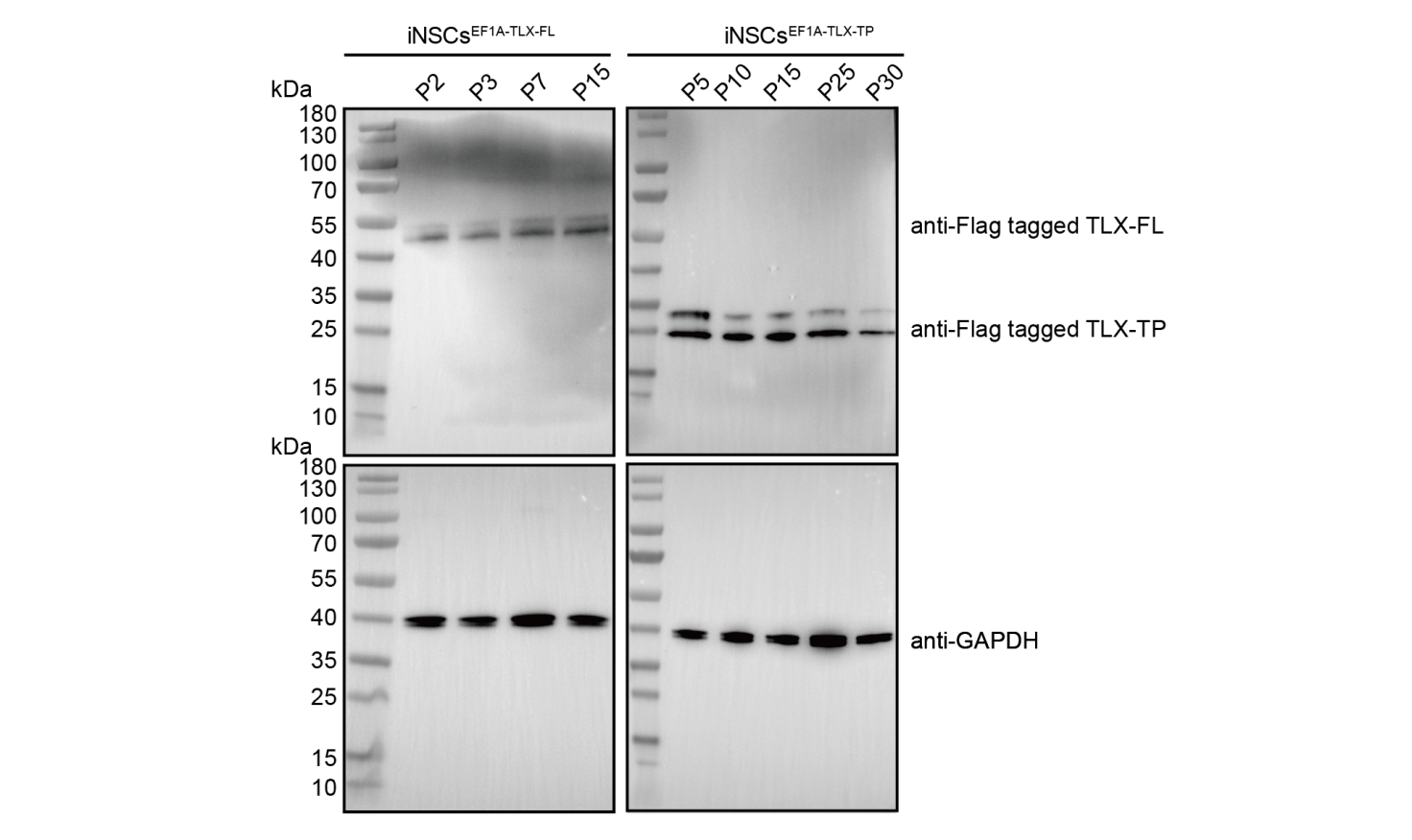


**Fig. S4** Expression of Flag and GAPDH in different passages of iNSCs^EF1a-TLX-FL/TP^ detected by Western blot.


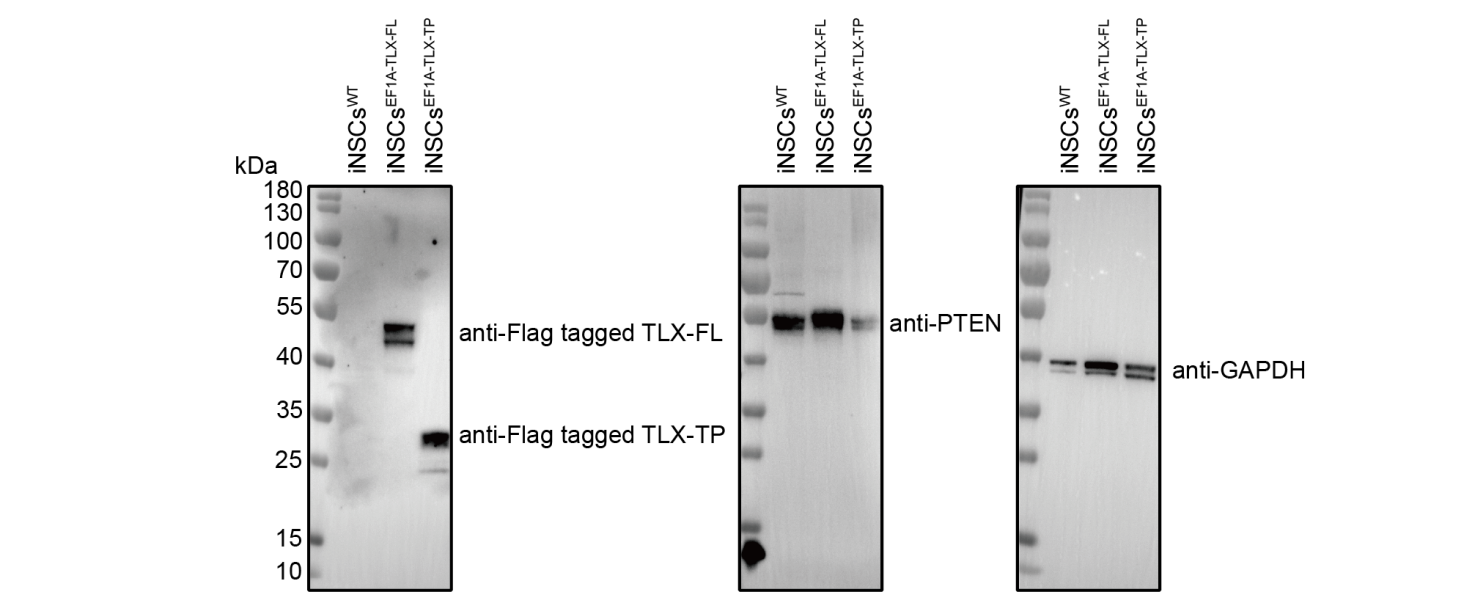


**Fig. S5** Protein expressions of Flag, PTEN, and GAPDH determined by Western blot.


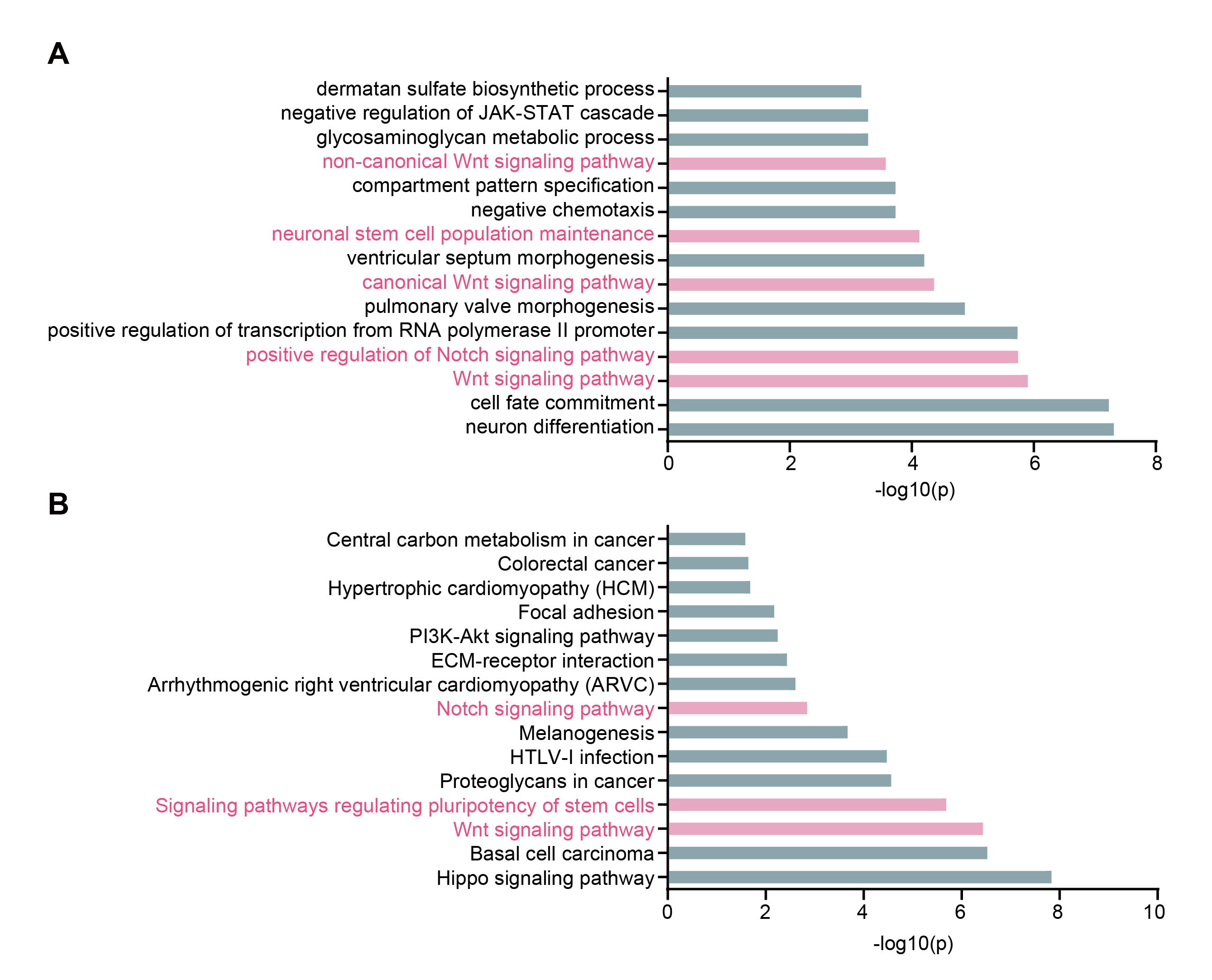


**Fig. S6** A) GO analysis, and B) KEGG analysis of differential gene expression profiles of iNSCs^TLX-TP^ versus iNSCs^TLX-FL^ in terms of biological processes.


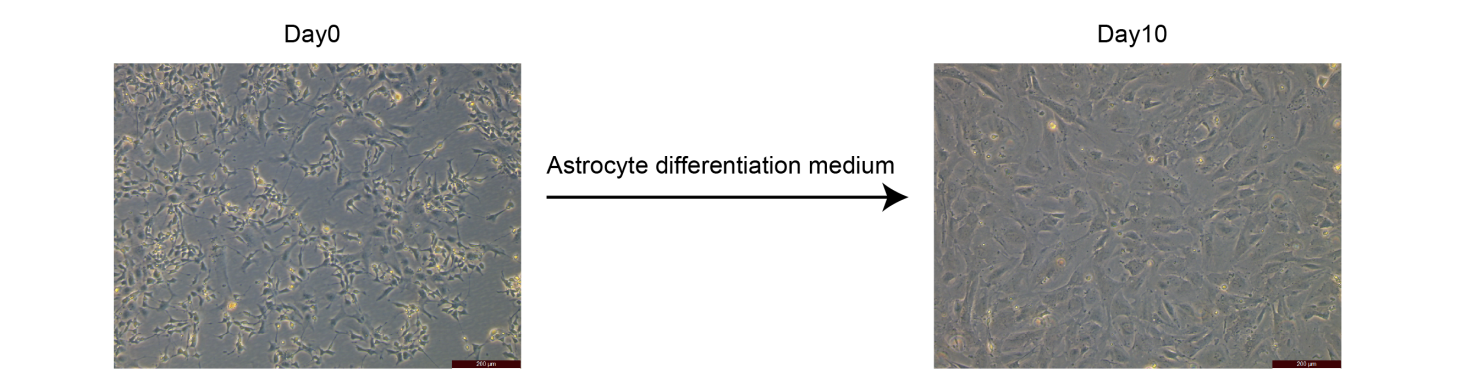


**Fig. S7** Cellular apoptosis during induction of iNSCs^TLX-TP^ differentiation into astrocytes.


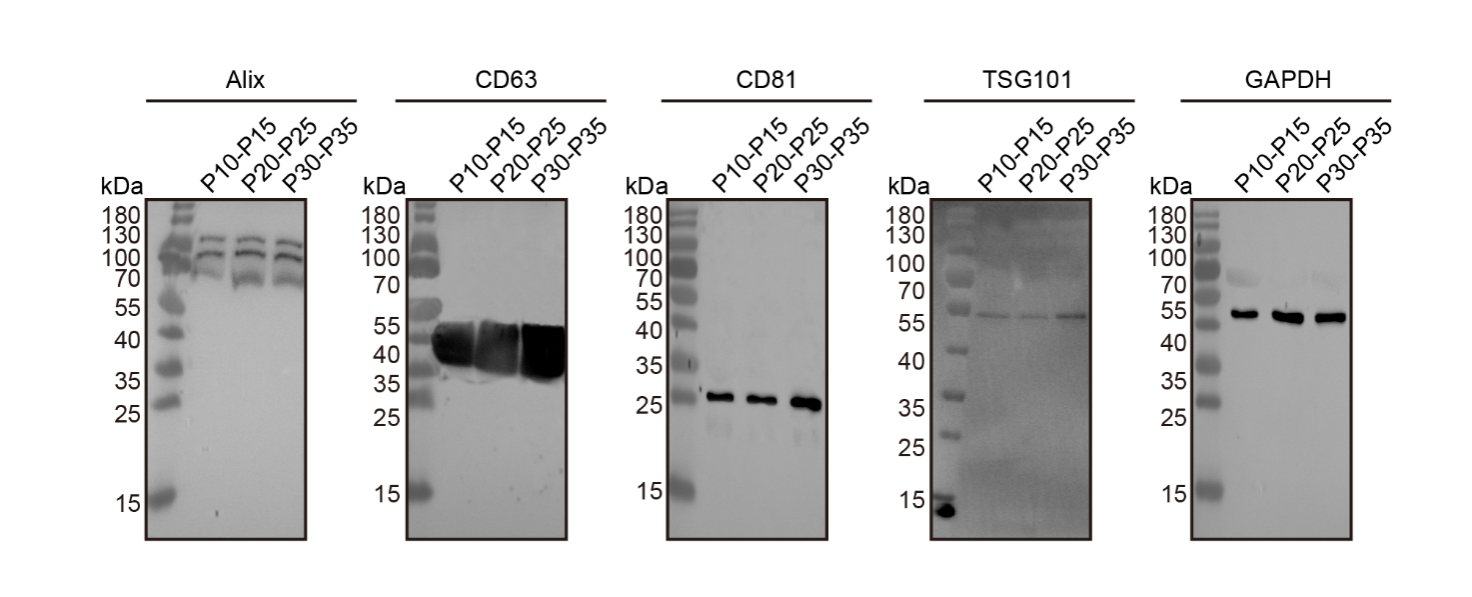


**Fig. S8** Immunoblotting of different batches iNSC-EVs for Alix, CD63, CD81, TSG101 and GAPDH expression.


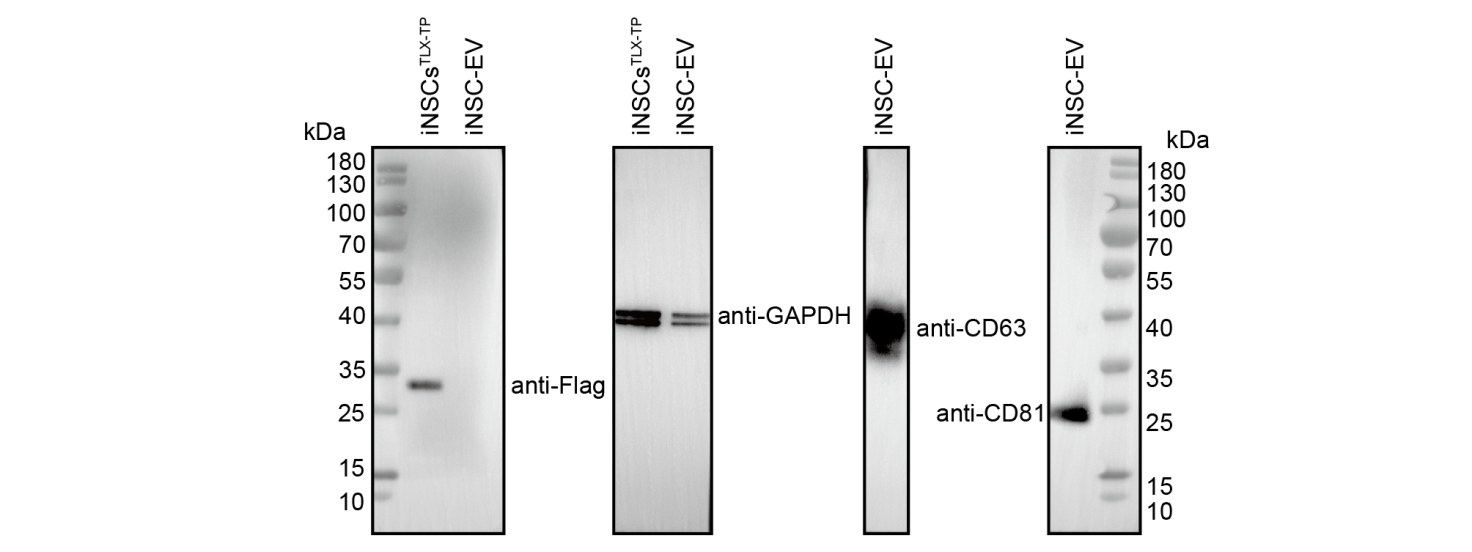


**Fig. S9** Immunoblotting of iNSCs^TLX-TP^ or purified iNSC-EVs for Flag, GAPDH, CD63, and CD81 expressions.


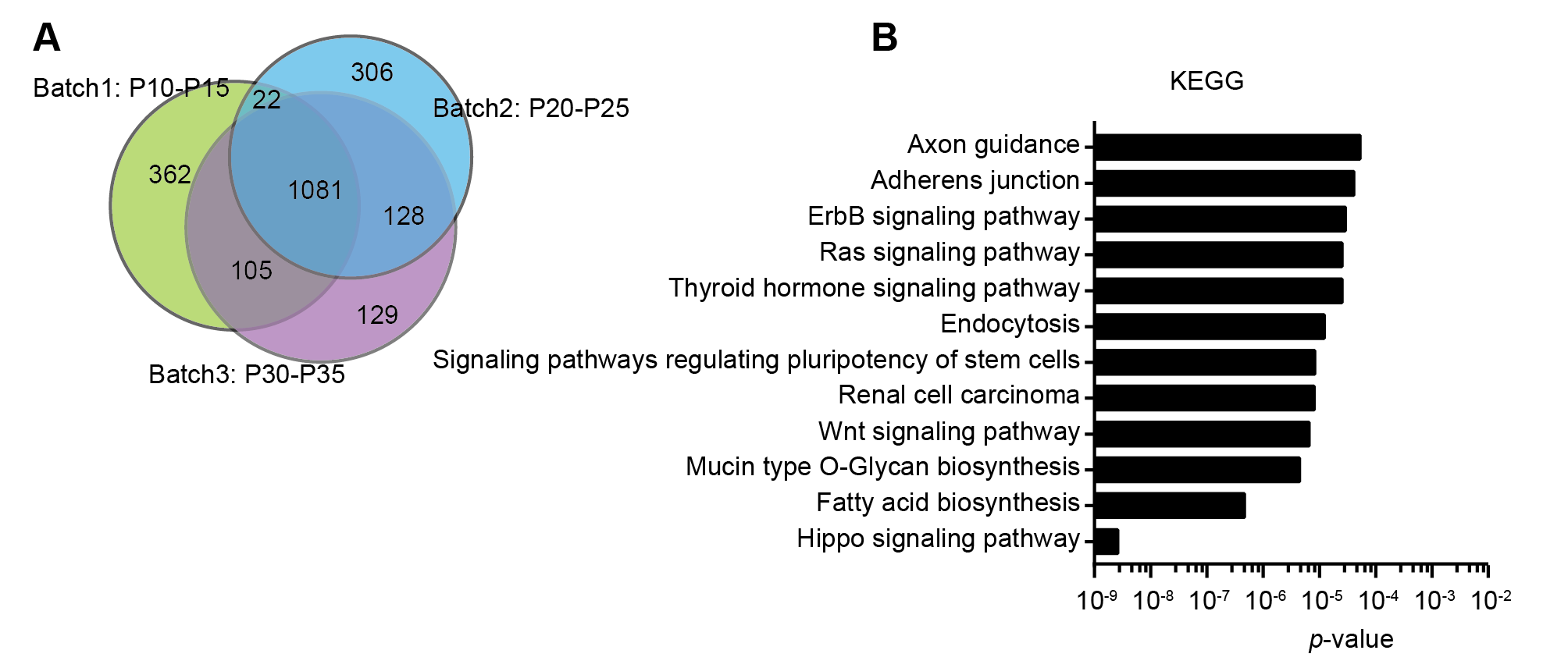


**Fig. S10** The results of transcriptomic analysis of three batches iNSC-EVs samples. A) A Venn diagram showing that about 70% of the iNSC-EVs miRNA population were shared among the three iNSC-EVs batches. B) KEGG analysis of 1081 common miRNA.
