# Supplementary material for: Stable expression of a truncated TLX variant drives differentiation of induced pluripotent stem cells into self-renewing neural stem cells for production of extracellular vesicles
Source: Stem Cell Res Ther. 2022 Sep 2;13:436. doi: 10.1186/s13287-022-03131-4 (PMC9438273; doi:10.1186/s13287-022-03131-4)
Supplement: Supplementary file 2 — Additional file 2 Table S1: Comparison of transcriptome profiles of iPSCsTLX-FL/TP and iPSCsWT. Table S2: List of media tested for iPSCsTLX-FL/TP differentiation. Table S3: List of media tested for iNSCsTLX-FL/TP expansion. Table S4: Differential gene expressions detected by RNA-sequencing. Table S5: NanoFCM analysis results of the media used in iNSCs culture. Table S6: The 25 most relevant pathways are overrepresented in the Reactome analysis. Table S7: Top 100 abundant miRNAs in iNSC-EVs. Table S8: Enriched KEGG pathways with functional categories related to the CNS. Table S9: TLX-targeting miRNAs detected in RNA-sequencing from iPSCsTLX-FL/TP and iNSCsTLX-FL/TP. Table S10: List of primers. [file 13287_2022_3131_MOESM2_ESM.docx]

Stable expression of a truncated TLX variant drives differentiation of induced pluripotent stem cells into self-renewing neural stem cells for production of extracellular vesicles

Mingzhi Xu^a^ , Gang Chen^a^ , Yanan Dong^a^ , Shensi Xiang^a^, Miaomiao Xue^a^ , Yongxue Liu^b^, Haijing Song^c*^, Haifeng Song^a*^, Yi Wang^a*^

**Additional file 2**

**Table S1** Comparison of transcriptome profiles of iPSCs^TLX-FL/TP^ and iPSCs^WT^.

Comparison of transcriptome profiles of iPSCs^TLX-FL^ and iPSCs^WT^.

| Gene name | RPKM | | GFOLD(0.01) |
| --- | --- | --- | --- |
|  | iPSCsWT | iPSCsTLX-FL |  |
| XIST | 28.742 | 0.108 | -7.889 |
| TSIX | 7.068 | 0.029 | -7.655 |
| NLRP2 | 14.609 | 0.215 | -5.845 |
| SIX6 | 4.508 | 0.289 | -3.593 |
| LRRC61 | 7.300 | 0.857 | -2.922 |
| NPTX1 | 6.813 | 1.050 | -2.659 |
| ALPPL2 | 7.630 | 1.852 | -1.950 |
| GDF3 | 7.352 | 1.981 | -1.708 |
| HMX1 | 4.502 | 1.223 | -1.707 |
| ZNF560 | 0.848 | 3.863 | 1.705 |
| RN7SL410P | 0.808 | 4.253 | 1.062 |
| FOS | 3.880 | 45.133 | 3.254 |
| ZNF728 | 0.103 | 3.778 | 3.962 |
| NR2E1 | 0.000 | 38.388 | 9.890 |

Comparison of transcriptome profiles of iPSCs^TLX-TP^ and iPSCs^WT^.

| Gene name | RPKM | | GFOLD(0.01) |
| --- | --- | --- | --- |
|  | iPSCs^WT^ | iPSCs^TLX-TP^ |  |
| XIST | 28.742 | 0.021 | -9.404 |
| TSIX | 7.068 | 0.008 | -8.616 |
| ALPPL2 | 7.630 | 0.186 | -4.386 |
| NLRP2 | 14.609 | 1.314 | -3.010 |
| SIX3 | 7.373 | 0.858 | -2.482 |
| ECEL1P2 | 3.816 | 0.476 | -2.166 |
| TXNIP | 32.551 | 6.048 | -2.023 |
| SOX21 | 6.340 | 1.220 | -1.790 |
| SDR42E1 | 0.888 | 3.155 | 1.804 |
| RN7SL4P | 2.308 | 8.419 | 1.518 |
| APLN | 0.939 | 3.435 | 1.859 |
| ZNF560 | 0.848 | 3.566 | 2.027 |
| MTND1P23 | 9.618 | 43.457 | 2.194 |
| RN7SL410P | 0.808 | 4.006 | 1.403 |
| RPS4Y1 | 0.000 | 5.142 | 6.713 |
| NR2E1 | 0.000 | 27.118 | 9.830 |

**Table S2** List of media tested for iPSCs^TLX-FL/TP^ differentiation.

| Name | Component | | | | Score |
| --- | --- | --- | --- | --- | --- |
|  | Brand | Medium | Supplement | other | iPSCs differentiation |
| NGD | thermo fisher | Neurobasal |  |  | 2 |
|  | thermo fisher |  | Glutamax |  |  |
|  | thermo fisher |  | Sodium pyruvate |  |  |
|  | thermo fisher |  | Pen/strep |  |  |
|  | Sigma |  | NaCI |  |  |
|  | Sigma |  | BSA |  |  |
|  | thermo fisher |  | B27 |  |  |
|  | thermo fisher |  | N2 |  |  |
| NGD-F |  | NGD |  |  | 4 |
|  | Sino biological |  |  | FGF2 |  |
| NGD-C |  | NGD |  |  | 0 |
|  | MCE |  |  | Coumpound C |  |
|  | Solarbio |  |  | Insulin |  |
|  | Sino biological |  |  | FGF2 |  |
| NGD-L |  | NGD |  |  | 0 |
|  | Sino biological |  |  | LIF |  |
| NGD-FI |  | NGD |  |  | 1 |
|  | Sino biological |  |  | FGF2 |  |
|  | Solarbio |  |  | Insulin |  |
| NGD-I |  | NGD |  |  | 5 |
|  | Solarbio |  |  | Insulin |  |
| NGD-FIL |  | NGD |  |  | 3 |
|  | Sino biological |  |  | FGF2 |  |
|  | Sino biological |  |  | LIF |  |
|  | Solarbio |  |  | Insulin |  |
| SD | Nuwacell | Epic |  |  | 0 |
|  | MCE |  |  | DMH-1 |  |
|  | MCE |  |  | SB431542 |  |
| LSL | Nuwacell | Epic |  |  | 0 |
|  | MCE |  |  | LDN |  |
|  | MCE |  |  | SB431542 |  |
|  | Sino biological |  |  | LIF |  |
| LSDL | Nuwacell | Epic |  |  | 0 |
|  | MCE |  |  | LDN |  |
|  | MCE |  |  | SB431542 |  |
|  | MCE |  |  | DMH1 |  |
|  | Sino biological |  |  | LIF |  |
| NIM | thermo fisher | DMEM/F12 |  |  | 0 |
|  | thermo fisher |  |  | N2 |  |
|  | thermo fisher |  |  | Glutamax |  |
|  | thermo fisher |  |  | NEAA |  |
|  | MCE |  |  | Heparin |  |
| NIM++ | thermo fisher | DMEM/F12 |  |  | 0 |
|  | thermo fisher |  |  | N2 |  |
|  | thermo fisher |  |  | Glutamax |  |
|  | thermo fisher |  |  | NEAA |  |
|  | MCE |  |  | Heparin |  |
|  | Sino biological |  |  | FGF2 |  |
|  | Sino biological |  |  | EGF2 |  |

**Table S3** List of media tested for iNSCs^TLX-FL/TP^ expansion.

| Name | Component | | | | Score |
| --- | --- | --- | --- | --- | --- |
|  | Brand | Medium | Supplement | other | iNSCs self-renewal |
| NGD-I | thermo fisher | Neurobasal |  |  | 3 |
|  | thermo fisher |  | Glutamax |  |  |
|  | thermo fisher |  | Sodium pyruvate |  |  |
|  | thermo fisher |  | Pen/strep |  |  |
|  | Sigma |  | NaCI |  |  |
|  | Sigma |  | BSA |  |  |
|  | thermo fisher |  | B27 |  |  |
|  | thermo fisher |  | N2 |  |  |
|  | Solarbio |  |  | Insulin |  |
| NIM | thermo fisher | DMEM/F12 |  |  | 2 |
|  | thermo fisher |  | N2 |  |  |
|  | thermo fisher |  | Glutamax |  |  |
|  | thermo fisher |  | NEAA |  |  |
|  | MCE |  |  | Heparin |  |
| NIM++ | thermo fisher | DMEM/F12 |  |  | 4 |
|  | thermo fisher |  | N2 |  |  |
|  | thermo fisher |  | Glutamax |  |  |
|  | thermo fisher |  | NEAA |  |  |
|  | MCE |  |  | Heparin |  |
|  | Sino biological |  |  | FGF2 |  |
|  | Sino biological |  |  | EGF2 |  |
| NEM | thermo fisher | DMEM/F12 |  |  | 5 |
|  | thermo fisher | Neurobasal |  |  |  |
|  | thermo fisher |  | NEM supplement |  |  |

**Table S4** Differential gene expressions detected by RNA-sequencing.

| Effect of TLX | Gene | FPKM | | GFOLD(0.01) |
| --- | --- | --- | --- | --- |
|  |  | iNSCs^TLX-FL^ | iNSCs^TLX-TP^ |  |
| Repress | PTEN | 11.180 | 6.640 | -0.673 |
| Repress | P21 | 4.036 | 29.100 | 2.694 |
| Repress | BMP4 | 14.640 | 1.820 | -2.786 |
| Promote | ASCL1 | 1.079 | 12.690 | 3.230 |
| Promote | Wnt7a | 0.000 | 3.970 | 6.657 |
| Promote | PAX6 | 0.185 | 3.008 | 3.703 |
| Promote | SOX2 | 6.190 | 141.760 | 4.384 |
| Promote | HES1 | 0.658 | 6.374 | 2.818 |

**Table S5** NanoFCM analysis results of the media used in iNSCs culture.

| Batch | Concentration (× 10^6^ p/mL) |
| --- | --- |
|  |  |
| 1 | 1.790 |
| 2 | 0.759 |
| 3 | 0.992 |
| 4 | 1.220 |
| 5 | 0924 |
| Mean ± SD | 1.137 ± 0.4008 |

**Table S6** The 25 most relevant pathways are over-represented in the Reactome analysis.


| Pathway name | Entities | | | Reactions |
| --- | --- | --- | --- | --- |
|  | Found/total | *p*-value | FDR | Found/total |
| Neurophilin interactions with VEGF and VEGFR | 3/4 | 1.11E-16 | 1.22E-15 | 4/4 |
| Translation | 71/339 | 1.11E-16 | 1.22E-15 | 56/99 |
| Neurons system development | 143/621 | 1.11E-16 | 1.22E-15 | 138/324 |
| Regulation of PTEN stability and activity | 37/74 | 1.11E-16 | 1.22E-15 | 7/13 |
| Regulation of apoptosis | 36/54 | 1.11E-16 | 1.22E-15 | 2/5 |
| Axon guidance | 141/585 | 1.11E-16 | 1.22E-15 | 134/298 |
| Regulation of expression of SLITs and ROBOs | 85/183 | 1.11E-16 | 1.22E-15 | 8/20 |
| Developmental biology | 194/1274 | 1.11E-16 | 1.22E-15 | 217/568 |
| Innate immune system | 182/1345 | 1.11E-16 | 1.22E-15 | 266/725 |
| Regulation of insulin-like growth factor transport and uptake by insulin-like growth factor binding proteins | 44/127 | 1.11E-16 | 1.22E-15 | 5/14 |
| Cellular responses to stress | 180/1005 | 1.11E-16 | 1.22E-15 | 151/450 |
| Metabolism of RNA | 128/789 | 1.11E-16 | 1.22E-15 | 62/187 |
| Immune system | 276/2698 | 1.11E-16 | 1.22E-15 | 528/1644 |
| Cellular responses to stimuli | 180/1023 | 1.11E-16 | 1.22E-15 | 151/481 |
| Signaling by hedgehog | 49/168 | 1.11E-16 | 1.22E-15 | 23/82 |
| Interleukin-1 signaling | 41/125 | 1.11E-16 | 1.22E-15 | 16/59 |
| PTEN regulation | 43/171 | 1.11E-16 | 1.22E-15 | 15/56 |
| Signaling by WNT | 58/332 | 1.11E-16 | 1.22E-15 | 111/157 |
| Deregulated CDK5 triggers multiple neurodegenerative pathways in Alzheimer’s disease models | 10/30 | 1.11E-16 | 1.22E-15 | 9/22 |
| NCAM signaling for neurite out-growth | 23/70 | 1.11E-16 | 1.22E-15 | 19/23 |
| Translation initiation complex formation | 31/62 | 1.11E-16 | 1.22E-15 | 2/2 |
| GTP hydrolysis and joining of the 60S ribosomal subunit | 55/120 | 1.11E-16 | 1.22E-15 | 3/3 |
| Signaling by NOTCH | 42/250 | 1.11E-16 | 1.22E-15 | 20/154 |
| Diseases of the neuronal system | 13/31 | 1.11E-16 | 1.22E-15 | 7/17 |
| p53-independent DNA damage response | 36/54 | 1.11E-16 | 1.22E-15 | 2/4 |

**Table S7** Top 100 abundant miRNAs in iNSC^TLX-TP^-EVs.

| #1-25 | #26-50 | #51-75 | #76-100 |
| --- | --- | --- | --- |
| hsa-miR-4652-3p | hsa-miR-668-3p | hsa-miR-5698 | hsa-miR-558 |
| hsa-miR-196a-1-3p | hsa-miR-6793-5p | hsa-let-7a-2-3p | hsa-miR-4653-3p |
| hsa-miR-3153 | hsa-miR-127-3p | hsa-miR-148b-3p | hsa-miR-6790-3p |
| hsa-miR-5197-3p | hsa-miR-3972 | hsa-miR-6090 | hsa-miR-4462 |
| hsa-miR-6131 | hsa-miR-138-2-3p | hsa-miR-6514-5p | hsa-miR-3621 |
| hsa-miR-6073 | hsa-miR-3613-3p | hsa-miR-6836-3p | hsa-miR-1234-3p |
| hsa-miR-4771 | hsa-miR-3679-3p | hsa-miR-10396b-5p | hsa-miR-181a-5p |
| hsa-miR-4709-5p | hsa-miR-4655-5p | hsa-miR-6867-5p | hsa-miR-1237-5p |
| hsa-miR-8062 | hsa-miR-1469 | hsa-miR-92a-3p | hsa-miR-6732-3p |
| hsa-miR-1246 | hsa-miR-651-3p | hsa-miR-885-5p | hsa-miR-6787-5p |
| hsa-miR-6847-5p | hsa-miR-1290 | hsa-miR-582-3p | hsa-miR-6728-5p |
| hsa-miR-2681-3p | hsa-miR-3960 | hsa-miR-671-3p | hsa-miR-542-3p |
| hsa-miR-615-3p | hsa-miR-6516-5p | hsa-miR-8058 | hsa-miR-4677-5p |
| hsa-miR-6513-5p | hsa-miR-6074 | hsa-miR-4634 | hsa-miR-5585-3p |
| hsa-miR-8069 | hsa-miR-218-2-3p | hsa-miR-6511a-5p | hsa-miR-433-5p |
| hsa-miR-1292-3p | hsa-miR-7151-3p | hsa-miR-4713-5p | hsa-miR-6855-5p |
| hsa-miR-92a-1-5p | hsa-miR-197-3p | hsa-miR-887-3p | hsa-miR-942-3p |
| hsa-miR-6756-3p | hsa-miR-302a-5p | hsa-miR-4516 | hsa-miR-3614-3p |
| hsa-miR-122-5p | hsa-miR-320e | hsa-miR-1273c | hsa-miR-4665-3p |
| hsa-miR-16-5p | hsa-miR-4669 | hsa-miR-3686 | hsa-miR-618 |
| hsa-miR-510-3p | hsa-miR-4741 | hsa-miR-6746-3p | hsa-miR-7847-3p |
| hsa-miR-764 | hsa-miR-125a-3p | hsa-miR-6724-5p | hsa-miR-6794-3p |
| hsa-miR-6831-3p | hsa-miR-3692-5p | hsa-miR-4458 | hsa-miR-2113 |
| hsa-miR-10400-5p | hsa-miR-4786-5p | hsa-miR-1208 | hsa-miR-342-3p |
| hsa-miR-664a-5p | hsa-miR-135b-3p | hsa-miR-184 | hsa-miR-7108-5p |

**Table S8** Enriched KEGG pathways with functional categories related to the CNS.

| Pathway | *p* value | # genes | miRNA | |
| --- | --- | --- | --- | --- |
|  |  |  | Number | Names |
| Fatty acid biosynthesis | 4.09E-10 | 4 | 1 | miR-16-5p |
| Signaling pathways regulating pluripotency of stem cells | 2.11E-06 | 76 | 6 | miR-1246, miR-92a-1-5p, miR-92a-1-5p, miR-138-2-3p,miR-3613-3p, miR-1290 |
| Hippo signaling pathway | 2.65E-04 | 79 | 4 | miR-16-5p, miR-3613-3p, miR-4655-5p, miR-651-3p |
| ECM-receptor interaction | 1.08E-03 | 25 | 7 | miR-6513-5p, miR-92a-1-5p, miR-6756-3p, miR-510-3p, miR-651-3p, miR-6516-5p, miR-6074 |
| Lysine degradation | 0.027 | 12 | 6 | miR-6513-5p, miR-668-3p, miR127-3p, miR-1290, miR-6516-5p, miR-218-2-3p |
| Mucin type O-Glycan biosynthesis | 0.039 | 6 | 4 | miR-5197-3p, miR-6847-5p, miR-8069, miR-122-5p |
| Glycosphingolipid biosynthesis - lacto and neolacto series | <1E-3.25 | 14 | 7 | miR-1246, miR-6513-5p, miR-764, miR-3972, miR-3972, miR-6516-5p, miR-6516-5p |

**Table S9** TLX-targeting miRNAs detected in RNA-sequencing from iPSCs^TLX-FL/TP^ and iNSCs^TLX-FL/TP^.

| miRNA Name | iPSCs^TLX-FL^ | iNSCs^TLX-FL^ | iPSCs^TLX-TP^ | iNSCs^TLX-TP^ |
| --- | --- | --- | --- | --- |
| [hsa-miR-205-3p](http://mirdb.org/cgi-bin/mature_mir.cgi?name=hsa-miR-205-3p) | 1.0566 | 0.0000 | 0.6250 | 0.0111 |

**Table S10** List of primers.

| Primer | Sequence |
| --- | --- |
| *GAPDH-F* | GGAGCGAGATCCCTCCAAAAT |
| *GAPDH-R* | GGCTGTTGTCATACTTCTCATGG |
| *SOX2-F* | GCCGAGTGGAAACTTTTGTCG |
| *SOX2-R* | GGCAGCGTGTACTTATCCTTCT |
| *OCT4-F* | CTTGAATCCCGAATGGAAAGGG |
| *OCT4-R* | GTGTATATCCCAGGGTGATCCTC |
| *NANOG-F* | TTTGTGGGCCTGAAGAAAACT |
| *NANOG-R* | AGGGCTGTCCTGAATAAGCAG |
| *NR2E1-F* | GCCGTTCCTACACATAGTGGTTCTG |
| *NR2E1-R* | TTTCCAAAGCGACAGGGTTGAGTG |
| *PTEN-F* | TGGATTCGACTTAGACTTGACCT |
| *PTEN-R* | GGTGGGTTATGGTCTTCAAAAGG |
| *p21-F* | TGTCCGTCAGAACCCATGC |
| *p21-R* | AAAGTCGAAGTTCCATCGCTC |
